# Incorporating substrate sequence motifs and spatial amino acid composition to identify kinase-specific phosphorylation sites on protein three-dimensional structures

**DOI:** 10.1186/1471-2105-14-S16-S2

**Published:** 2013-10-22

**Authors:** Min-Gang Su, Tzong-Yi Lee

**Affiliations:** 1 Department of Computer Science and Engineering, Yuan Ze University, Taoyuan 320, Taiwan

**Keywords:** phosphorylation, protein kinase, three-dimensional structure, spatial amino acid composition

## Abstract

**Background:**

Protein phosphorylation catalyzed by kinases plays crucial regulatory roles in cellular processes. Given the high-throughput mass spectrometry-based experiments, the desire to annotate the catalytic kinases for *in vivo *phosphorylation sites has motivated. Thus, a variety of computational methods have been developed for performing a large-scale prediction of kinase-specific phosphorylation sites. However, most of the proposed methods solely rely on the local amino acid sequences surrounding the phosphorylation sites. An increasing number of three-dimensional structures make it possible to physically investigate the structural environment of phosphorylation sites.

**Results:**

In this work, all of the experimental phosphorylation sites are mapped to the protein entries of Protein Data Bank by sequence identity. It resulted in a total of 4508 phosphorylation sites containing the protein three-dimensional (3D) structures. To identify phosphorylation sites on protein 3D structures, this work incorporates support vector machines (SVMs) with the information of linear motifs and spatial amino acid composition, which is determined for each kinase group by calculating the relative frequencies of 20 amino acid types within a specific radial distance from central phosphorylated amino acid residue. After the cross-validation evaluation, most of the kinase-specific models trained with the consideration of structural information outperform the models considering only the sequence information. Furthermore, the independent testing set which is not included in training set has demonstrated that the proposed method could provide a comparable performance to other popular tools.

**Conclusion:**

The proposed method is shown to be capable of predicting kinase-specific phosphorylation sites on 3D structures and has been implemented as a web server which is freely accessible at http://csb.cse.yzu.edu.tw/PhosK3D/. Due to the difficulty of identifying the kinase-specific phosphorylation sites with similar sequenced motifs, this work also integrates the 3D structural information to improve the cross classifying specificity.

## Introduction

Protein phosphorylation catalyzed by kinases plays crucial regulatory roles in many essential cellular processes including cellular regulation, cellular signal pathways, metabolism, growth, differentiation, and membrane transport [1]. It has been estimated that one-third to one-half of all proteins are phosphorylated in a eukaryotic cell [2] and around half of kinome are disease- or cancer-related by chromosomal mapping [3]. Mass spectrometry-based identifications of phosphorylation sites on substrates *in vivo *and *in vitro *are the foundation of understanding the mechanisms of phosphorylation dynamics and important for the biomedical drug design [4]. However, the effort to experimentally verify the catalytic kinases remains time-consuming, labor-intensive, and expensive. Thus, many researches are undertaken to develop a computational method for the identification of kinase-specific phosphorylation sites, including NetPhosK [5], Scansite 2.0 [6], PredPhospho [7], GPS [8], PlantPhos [9], PPSP [4], MetaPredPS [10], NetPhorest [11] and KinasePhos [12-14]. The summary information of the previously developed phosphorylation site prediction methods is listed in Table S1 (Additional File 1). Particularly, Linding *et al. *[15] have proposed an excellent method, namely NetworKIN, that augments motif-based predictions with the network context of kinases and phosphoproteins. With most of the existing phosphorylation site prediction tools requiring prior knowledge of experimentally verified substrates and its kinase, a method is developed to be able to predict kinase-specific phosphorylation sites based solely on protein sequence [16].

Although over 20 methods have been developed for the accurate prediction of kinase-specific phosphorylation sites, most of them rely solely on the local amino acid sequence surrounding the phosphorylated sites. Blom *et al. *[17] were the first to propose a method with limited data for sequence and structure-based prediction of protein phosphorylation sites in eukaryotes. While one-dimensional amino acid sequence was observed to harbor most of the predictive power, Predikin [18] has proposed a method that applied the structure-based information for improving the prediction of phosphorylation sites in proteins. With an increasing interest in the structural environment of protein phosphorylation sites, Phospho3D database [19,20] was proposed for characterizing the structural properties of phosphorylation sites on three-dimensional (3D) structures. Additionally, Phos3D [21] has extracted 3D-signature motifs from 750 experimentally verified phosphorylation sites with 3D structures available in Protein Data Back (PDB) [22] and applied them to implement a web server for structure-based detection of phosphorylation sites.

With the desire to investigate the spatial environment of phosphorylation sites, all of the experimental phosphorylation sites are mapped to the PDB protein entries using sequence identity. In this work, the linear motifs are combined with the information of spatial amino acid composition, which is a new scheme for encoding a 3D structure fragment of phosphorylated sites, to identify kinase-specific phosphorylation sites on 3D structures. Moreover, an independent testing set which is blind to the cross-validation process has been generated for the evaluation of stability and reliability of the proposed method. To investigate the effect of including structural characteristics for identifying kinase-specific phosphorylation sites with similar substrate motifs, the cross classifying specificities among the kinase-specific models are evaluated.

## Materials and methods

**Figure 1:**
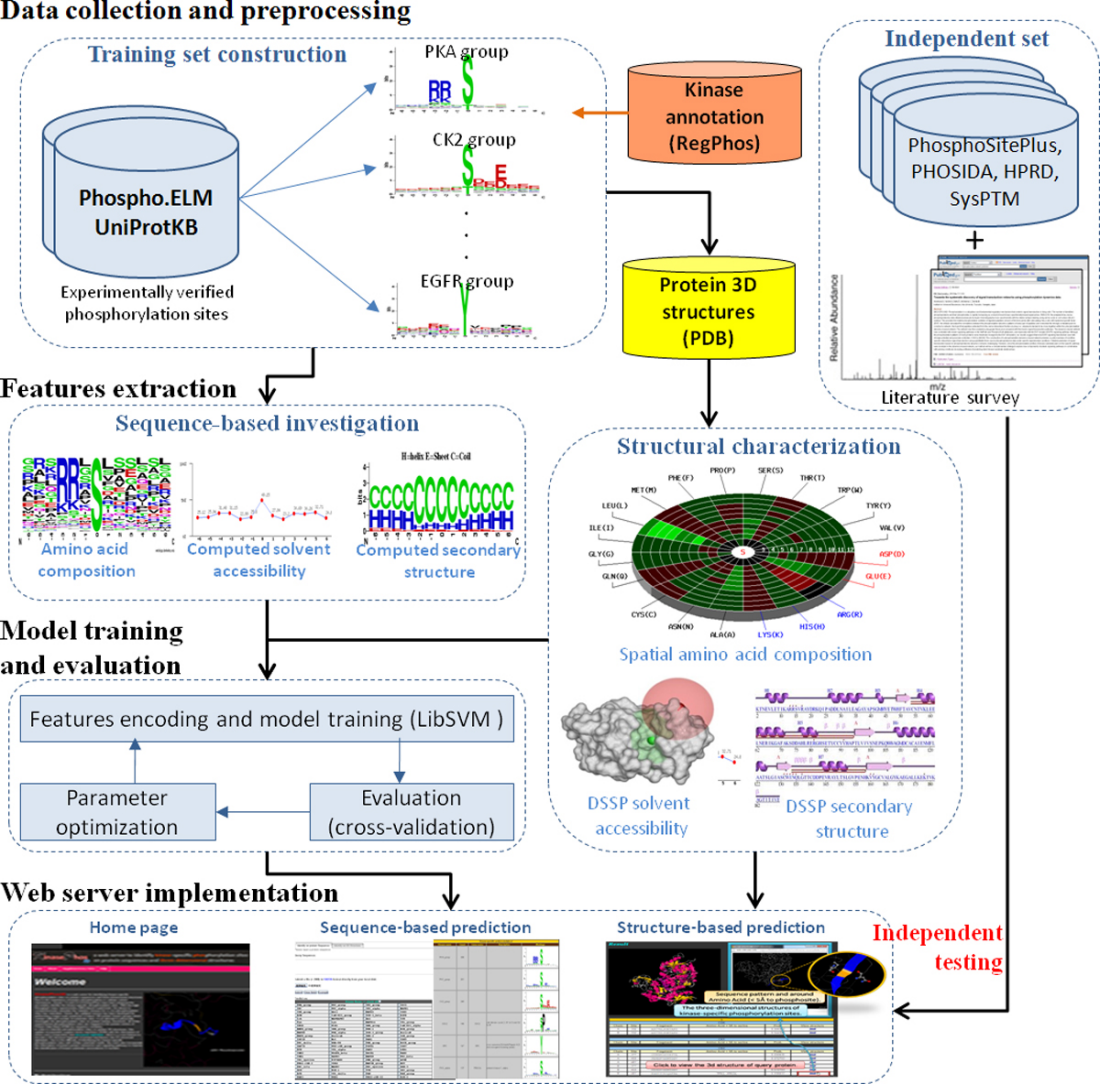
**System flow of the proposed method**.

Figure 1 depicts the system flow of the proposed method, including data collection and preprocessing, sequence-based investigation, structural characterization, model training and evaluation, and independent testing. The experimentally verified phosphorylation sites are mainly extracted from dbPTM [23,24] which has integrated the data from version 9.0 of Phospho.ELM [25], release 20120711 of UniProtKB [26], release 20120730 of PhosphoSitePlus [27], version 1.0 of PHOSIDA [28], version 1.1 of SysPTM [29] and version 9.0 of HPRD [30]. In this work, the data set extracted from Phospho.ELM and UniProtKB is regarded as the training set for sequential and structural investigation of phosphorylated substrate sites. After removing the redundant sites between Phospho.ELM and UniProtKB, the number of serine (S), threonine (T), and tyrosine (Y) substrate sites are 98376, 25269, and 15188, respectively, as given in Table 1. According to the annotations of kinase families extracted from KinBase [3] and RegPhos [31], the substrate sites of protein phosphorylation could be further categorized into more than 200 kinase groups. Table S2 (in Additional File 1) summarizes the data statistics of 122 kinase groups containing more than 10 substrate sites in the training set.

**Table 1:** **Data statistics of experimentally verified phosphorylation sites in each resource**.

Data set	Data Resource	Version	Number of phosphorylation sites			Number of phosphorylated proteins
				
			S	T	Y	
**Training set**	Phospho.ELM	9.0	26,136	6,316	3,118	8,690
	
	UniProtKB	20120711	92,221	23,289	14,337	34,040
	
	**Combined (NR^1^)**	-	**98,376**	**25,269**	**15,188**	**35,047**

**Independent testing set**	PhosphoSitePlus	20120730	73,969	19,946	14,696	18,550
	
	PHOSIDA	1.0	7,391	1,300	278	2,212
	
	SysPTM	1.1	30,307	6,643	2,255	10,667
	
	HPRD	9.0	34,273	10,761	4,121	7,753
	
	**Combined (NR^1^)**	**-**	**97,753**	**27,421**	**16,531**	**23,813**

^1^NR, non-redundant.

As for classification, the prediction performance of the constructed models may be overestimated owing to the over-fitting of a training set. The experimental phosphorylation sites that collected from PhosphoSitePlus, PHOSIDA, SysPTM, and HPRD were regarded as the independent testing set. Additionally, about 500 kinase-specific phosphorylation sites manually curate from 200 research articles are included in the independent testing set.

### Sequence-based investigation of phosphorylation sites

Since the flanking sequences of the substrate sites (position 0) are graphically visualized as the entropy plots of sequence logo [32,33], the conservation of amino acids surrounding the phosphorylation sites could be easily observed [34]. The 13-mer sequences (from -6 to +6) of kinase-specific phosphorylation sites are extracted as the positive data of training sets, while all other residues (S, T and Y) in the phosphorylated proteins are regarded as the negative data. With reference to the method of SulfoSite [35], the positional weighted matrix (PWM), which specifies the relative frequency of amino acids surrounding substrate sites, was utilized in encoding the fragment sequences. A matrix of *m *× *w *elements was used to represent each residue of a training dataset, where *w *stands for the window size and *m *consists of 21 elements including 20 types of amino acids and one for terminal signal.

Besides the composition of flanking amino acids, the accessible surface area (ASA) and secondary structure (SS) around the phosphorylation sites were also investigated. Since most of the experimentally verified phosphorylation sites do not have corresponding three-dimensional structures in PDB, with reference to MASA [36], an effective tool, RVP-Net [37,38], was applied to compute the ASA value from the protein sequence. The full-length protein sequences with experimentally identified phosphorylation sites are inputted to RVP-Net to compute the ASA value of all of the residues. The ASA values of amino acids around the phosphorylation sites are extracted and normalized to be between zero and one. Additionally, PSIPRED [39] was employed to compute the secondary structure from the protein sequence. PSIPRED 2.0 achieved a mean Q_3 _score of 80.6% across all 40 submitted target domains without obvious sequence similarity to structures that are present in PDB; accordingly, PSIPRED has been ranked top out of 20 evaluated methods [40]. The output of PSIPRED is given in terms of "H," "E" and "C" which stand for helix, sheet and coil, respectively.

### Structural characterization of phosphorylation sites

In an attempt to study the spatial context of phosphorylation sites and evaluate its effectiveness for improving the predictive performance, all of the collected phosphorylation sites are mapped to the protein entries of Protein Data Bank (PDB) by sequence identity. It resulted in a total of 4508 phosphorylation sites (covering over 40 kinase groups) containing the protein 3D structures. DSSP [41] is then utilized to calculate the surface solvent accessibility and standardize the secondary structure of PDB entries with the mapped phosphorylation sites. Instead of the sequential amino acid composition (AAC), this work investigates the propensities for the different amino acid types to occur in the spatial vicinity of the phosphorylated sites. A spatial amino acid composition (Spatial AAC) is determined for each kinase groups by calculating the relative frequencies of 20 amino acid types within radial distances ranging from 3 to 12 Å from central phosphorylated amino acid residue. A radial cumulative propensity plot [21] was applied to display the spatial AAC. In order to identify the significant difference of spatial AAC between phosphorylation sites (positive data) and non-phosphorylation sites (negative data), a measurement of F-score [42,43] has been applied to calculate a statistical value for each radial distance. The F-score of the *i*th value of 11 radial distances is defined as:

(1) $$\text{F}-\text{score }\left(i\right)=\frac{{\left({{\bar{x}}_{i}}^{\left(+\right)}-{\bar{x}}_{i}\right)}^{2}+{\left({{\bar{x}}_{i}}^{\left(-\right)}-{\bar{x}}_{i}\right)}^{2}}{\frac{1}{n^{+}-1}\overset{n^{+}}{\underset{k=1}{\sum }}{\left(x_{k,i}^{\left(+\right)}-{{\bar{x}}_{i}}^{\left(+\right)}\right)}^{2}+\frac{1}{n^{-}-1}\overset{n^{-}}{\underset{k=1}{\sum }}{\left(x_{k,i}^{\left(-\right)}-{{\bar{x}}_{i}}^{\left(-\right)}\right)}^{2}}$$

where ${\bar{x}}_{i}$, ${{\bar{x}}_{i}}^{\left(+\right)}$ and ${{\bar{x}}_{i}}^{\left(-\right)}$ denote the average value of the *i*th distance value in whole, positive, and negative data sets, respectively; $n^{+}$ denotes the number of positive data set and $n^{-}$ denotes the number of negative data set; $x_{k,i}^{\left(+\right)}$ denotes the *i*th distance value of the *k*th positive instance, and $x_{k,i}^{\left(-\right)}$ denotes the *i*th distance value of the *k*th negative instance [42].

### Model training and evaluation

This work incorporates support vector machines (SVMs) with the sequential and structural features to generate the predictive models for the identification of kinase-specific phosphorylation sites. A public SVM library, namely LIBSVM [44], is applied for training the predictive models. The radial basis function (RBF) $K\left(S_{i},S_{j}\right)=\text{exp}\left(-\gamma {∥S_{i}-S_{j}∥}^{2}\right)$ is selected as the kernel function of SVM. Five-fold cross-validation is used to evaluate the predictive performance of the models trained from the large data sets such as PKA, PKC, CK2, and MAPK groups, while Jackknife cross-validation is applied for models trained from the data size smaller than 30 substrate sites. We balance the positive set and negative set and the sizes of positive data and negative data are equal during the cross-validation processes. The cross-validation is performed for ten times to obtain an average accuracy for each kinase group. The following measures of predictive performance of the trained models are defined: Precision (Pre) = TP/(TP+FP), Sensitivity (Sn) = TP/(TP+FN), Specificity (Sp) = TN/(TN+FP) and Accuracy (Acc) = (TP + TN)/(TP+FP+TN+FN), where TP, TN, FP and FN are true positive, true negative, false positive and false negative predictions, respectively. The models trained with various features that yield the highest accuracy in each kinase group are utilized to implement the prediction system and are further evaluated by independent testing set. For a meaningful comparison with other published tools, the ratio of data size between positive set and negative set is 1:2 [21].

## Results and discussion

### Sequential and structural characteristics of kinase-specific phosphorylation sites

**Figure 2:**
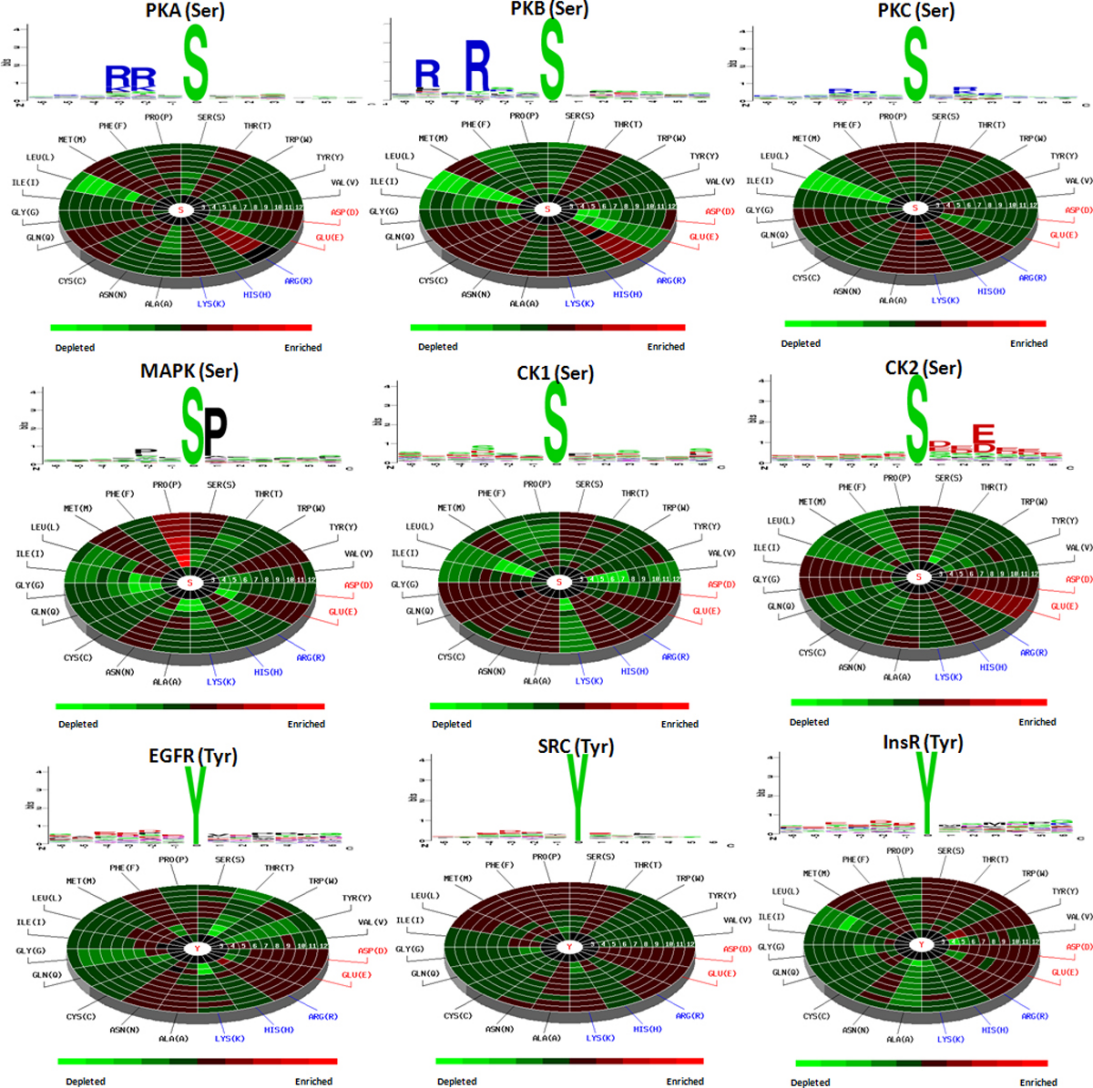
**Sequence logos and radial cumulative propensity plots of nine kinase-specific substrate groups**.

As the sequence logos given in Table S2 (Additional File 1), most of the kinase groups have conserved amino acids surrounding the phosphorylation sites. The solvent accessibility and secondary structure computed from a full-length protein sequence are also presented. With the comprehensive mapping between the collected phosphorylation data and PDB protein 3D structures, the spatial environment of phosphorylation sites was investigated in detail, as well as the sequential neighborhood. Figure 2 shows the sequence logos (sequential neighborhood) and radial cumulative propensity plots (spatial neighborhood) of nine well-known kinase-specific substrate groups. According to the observation from sequence logos, PKA and PKB have the significant enrichments of Arginine (R) and Lysine (K) in the sequential neighborhood of substrate sites, which is the hallmark sequence motif for AGC kinase families. The PKC group contains the slight enrichments of Arginine (R) and Lysine (K) around the substrate sites. However, the radial cumulative propensity plots present that there is an additional enrichment of amino acid residues in the spatial neighborhood. For instance, PKA exhibits the enrichments of Methionine (M), Glutamine (Q) and Aspartic acid (D) in the spatial neighborhood, accompanied by a remarkable depletion of Leucine (L) residue. The PKB group has the enrichments of Asparagine (N), Cysteine (C) and Threonine (T) in the spatial neighborhood, accompanied by the remarkable depletions of Glutamic acid (E) and L residues. For PKC group, there are the enrichments of Alanine (A) and Tyrosine (Y) in the spatial neighborhood, also accompanied by a remarkable depletion of L residue.

For MAPK group, there is a consistent enrichment of Proline (P) in sequential and spatial neighborhoods. Additionally, the enrichments of M and Y residues in spatial neighborhood are identified from the radial cumulative propensity plot. According to the sequence logo, there is no significant enrichment of amino acids for CK1 group. However, the radial cumulative propensity plot shows that there are slight enrichments of Histidine (H), E, A, N, C, Q, G and S residues in the spatial neighborhood, accompanied by the remarkable depletions of Valine (V), K and L residues. The CK2 group contains the consistent enrichments of D and E residues in sequential and spatial neighborhoods. According to the radial cumulative propensity plot, there are slight enrichments of Glycine (G), Isoleucine (I) and H residues in spatial neighborhood.

For tyrosine kinase families, EGFR, SRC and InsR groups have the enrichments of D and E residues in the sequential and spatial neighborhood. In particular, EGFR group has a significant depletion of T residue according to the radial cumulative propensity plot, but SRC and InsR groups are enriched in T residue instead. In summary, the radial cumulative propensity plot reveals spatial preferences of amino acids composition which cannot be identified by inspecting the sequence logo alone. In addition to the spatial preferences of amino acids composition, a summary list of structural characteristics, including spatial AAC, solvent accessibility and secondary structure, for 20 kinase-specific substrate groups which contain more than 10 substrate sites on 3D structures is illustrated in Table S3 (Additional File 1).

### Predictive performance of kinase-specific SVM models

For finding the best predictive performance of SVM models in each kinase-specific group, the SVM models trained with sequenced characteristics such as amino acid composition, solvent accessibility and secondary structure computed from protein sequence, positional weighted matrix are evaluated based on cross-validation. To obtain a stable performance for each kinase-specific prediction models, the cross-validation process is performed for ten times and the average sensitivity (Sn), specificity (Sp), and accuracy (Acc) of the SVM models are calculated as shown in Table S4 (Additional File 1). The overall cross-validation performance of SVM models trained with the hybrid combination of sequenced characteristics, whose average accuracy is close to 90.0%, is performing better than the SVM models trained with only amino acid composition. Additionally, the performance of independent testing for each kinase-specific model is also given in Table S4 (Additional File 1). Most of the SVM models have a predictive accuracy approaching to their cross-validation performance, while several kinase-specific SVM models trained with small data size of training set have an unstable predictive accuracy.

With the consideration of data sufficiency in structural investigation, the kinase-specific groups containing more than ten phosphorylation sites on 3D structures are studied in this work. Table 2 presents the cross-validation performance of kinase-specific SVM models trained with various features, including sequence-only information, structural information, and the combination of sequence and structural information. In general, the kinase-specific SVM models trained with structural information yield a better predictive accuracy than the SVM models trained with only sequence information. Additionally, the SVM models trained with the combination of sequence and structural characteristics were observed to perform at comparable or even slightly better performance levels compared to the SVM models trained with structural information. In summary, for all kinase-specific phosphorylation sites prediction, a consistent increase in performance was obtained suggesting that including 3D structural information does indeed improve the sensitivity and specificity.

**Table 2:** **Cross-validation evaluation of sequence and structure-based phosphorylation site predictions on 3D structures**.

Kinase group	Number of positive data	Number of negative data	Sequence-only			Structural information			Combination of sequence and structural information		
			
			Sn	Sp	Acc	Sn	Sp	Acc	Sn	Sp	Acc
**Phosphorylated Serine (pSer)**											

All serine data	1554	3108	61.4%	62.0%	61.8%	66.9%	68.1%	67.7%	**72.9%**	**71.1%**	**71.7%**

CDK	11	22	72.7%	81.8%	78.8%	**90.9%**	**86.8%**	**87.9%**	90.9%	86.8%	87.9%

CK1	10	20	20.0%	90.0%	66.7%	**100%**	**95.0%**	**96.7%**	100%	95.0%	96.7%

CK2	24	48	66.7%	87.5%	80.6%	87.5%	87.5%	87.5%	**91.7%**	**89.6%**	**90.3%**

MAPK	17	34	52.9%	94.1%	80.4%	76.5%	97.1%	90.2%	**82.4%**	**97.1%**	**92.2%**

PIKK	15	30	26.7%	83.3%	64.4%	**80.0%**	**86.7%**	**84.4%**	73.3%	83.3%	80.0%

PKA	56	112	79.1%	78.8%	78.9%	83.6%	84.3%	84.1%	**89.1%**	**91.4%**	**90.7%**

PKB	12	24	75.0%	66.7%	69.4%	75.0%	83.3%	80.6%	**83.3%**	**83.3%**	**83.3%**

PKC	50	100	77.3%	78.0%	77.8%	81.2%	80.0%	80.4%	**85.3%**	**86.0%**	**85.8%**

PKG	10	20	80.0%	80.0%	80.0%	**80.0%**	**85.0%**	**83.3%**	80.0%	85.0%	83.3%

PLK	10	20	60.0%	80.0%	73.3%	**70.0%**	**90.0%**	**83.3%**	70.0%	90.0%	83.3%

STE20	10	20	70.0%	75.0%	73.3%	**80.0%**	**90.0%**	**86.7%**	80.0%	90.0%	86.7%

**Phosphorylated Threonine (pThr)**											

All Threonine data	603	1206	60.9%	59.7%	60.1%	67.8%	67.2%	67.4%	**70.1%**	**72.5%**	**71.3%**

MAPK	13	26	**69.2%**	**76.9%**	**74.3%**	69.2%	76.9%	74.3%	69.2%	76.9%	74.3%

PKA	10	20	70.0%	90.0%	83.3%	80.0%	85.0%	83.3%	**80.0%**	**95.0%**	**90.0%**

PKC	13	26	61.5%	76.9%	71.8%	**69.2%**	**88.5%**	**82.1%**	69.2%	88.5%	82.1%

STE20	10	20	40.0%	95.0%	76.7%	70.0%	70.0%	70.0%	**70.0%**	**90.0%**	**80.0%**

**Phosphorylated Tyrosine (pTyr)**											

All tyrosine data	629	1258	62.0%	63.3%	62.8%	64.1%	63.4%	63.8%	**67.6%**	**68.6%**	**68.3%**

Abl	18	36	50.0%	88.9%	75.9%	**66.7%**	**80.6%**	**75.9%**	66.7%	80.6%	75.9%

EGFR	10	20	60.0%	80.0%	73.3%	**60.0%**	**95.0%**	**83.3%**	60.0%	95.0%	83.3%

InsR	15	30	73.3%	83.3%	80.0%	80.0%	80.0%	80.0%	**80.0%**	**90.0%**	**86.7%**

Src	57	114	77.2%	75.4%	76.0%	79.1%	83.3%	81.9%	**79.1%**	**84.9%**	**82.9%**

Syk	11	22	63.6%	90.9%	81.8%	72.7%	86.4%	81.8%	**72.7%**	**95.5%**	**87.9%**

Abbreviation: Sn, sensitivity; Sp, specificity; Acc, accuracy.

### Implementation of web-based prediction system

**Figure 3:**
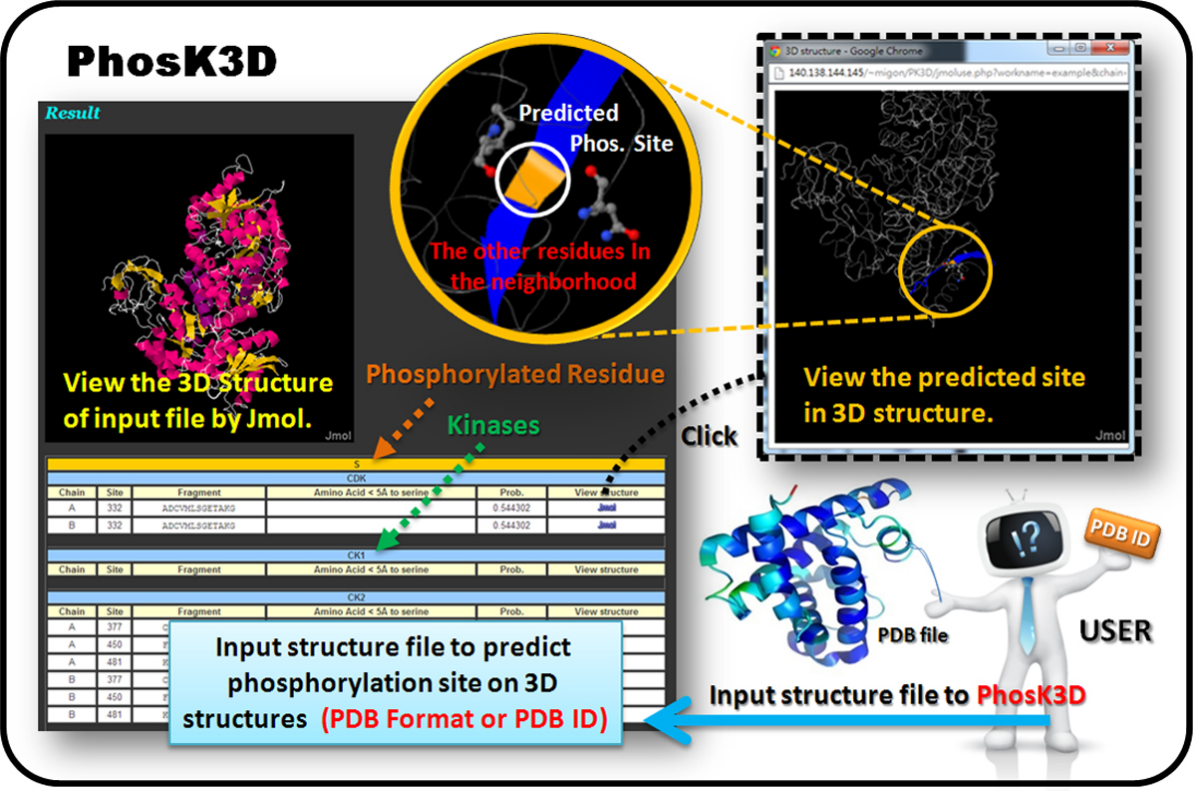
**The web interface of PhosK3D prediction system**. The PhosK3D locates the predictive phosphorylation sites and the involved catalytic protein kinases. In order to reveal the characteristics of the phosphorylation sites including the phosphorylated residues and surrounding sequences, the training set of phosphorylation sites and constructed sequence logos corresponding to each protein kinase are also provided graphically on the web interface. Additionally, users can download the predicted results with tab-delimited format for further analyses. Since a PDB ID or structure file is inputted to PhosK3D, the sequential neighborhood (blue) and spatial neighborhood (gray) of the predicted phosphorylation sites (orange) are provided to users. Moreover, the positively charged residues (blue) and negatively charged residues (red) surrounding the predicted phosphorylation sites are physically presented by Jmol viewer.

**Figure 4:**
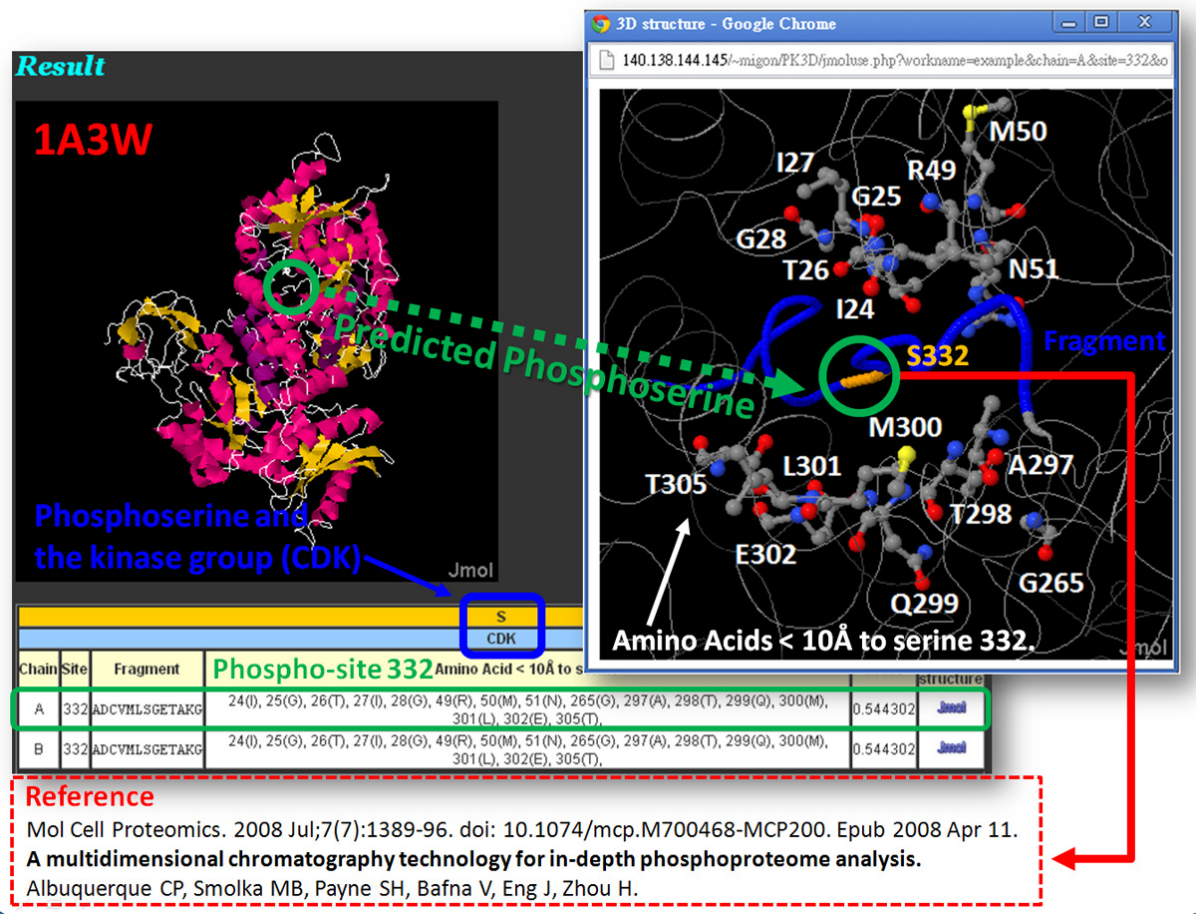
**A case study of phosphorylation sites prediction on the protein structure of Pyruvate kinase 1 (PDB ID: 1A3W)**.

**Figure 5:**
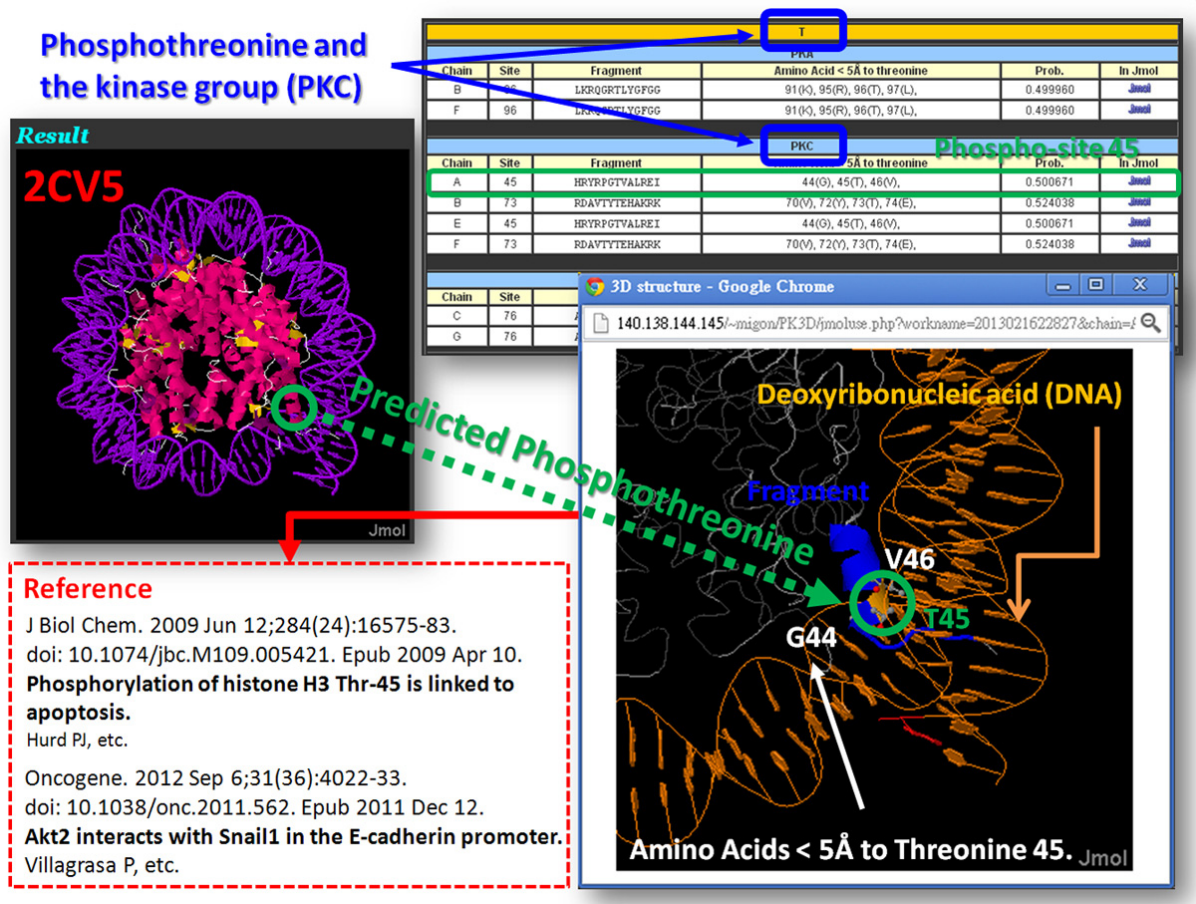
**A case study of phosphorylation sites prediction on protein structure of Histone (PDB ID:2CV5)**.

After evaluating the trained models for identifying kinase-specific phosphorylation sites, the SVM model yielding the highest predictive accuracy for each kinase group was utilized to implement the web-based prediction system. The system provides over 120 kinase-specific SVM models for performing a large-scale prediction on protein 3D structures. Users can submit their uncharacterized protein sequences and select the kinase-specific models for predicting phosphorylated Serine, Threonine, or Tyrosine. As presented in Figure 3, since a PDB ID or structure file is inputted to PhosK3D, the sequential and structural models will be integrated to identify the kinase-specific phosphorylation sites on the 3D structure. Moreover, the positively charged residues (K, R and H) and negatively charged residues (D and E) surrounding the predicted phosphorylation sites are physically presented as a surface view of Jmol viewer. Two case studies of kinase-specific phosphorylation sites prediction on protein 3D structures of Pyruvate kinase 1 (PDB ID: 1A3W) and Histone (PDB ID: 2CV5) are presented in Figure 4 and 5, respectively.

### Effect of including structural information for identifying kinase-specific phosphorylation sites with similar sequence motifs

As the sequence logos given in Table S2 (Additional File 1), it would be noticed that some of kinase groups have similar substrate motifs. For instance, several kinases (PKA, PKB, PKC, PKG, GRK, RSK,) of AGC family prefer to recognize the substrate sites with basic amino acids (Arginine, Lysine or Histidine) at positions of -2 or -3 relative to the phosphorylation sites (position 0). As given in Table S5 (Additional File 1), in order to assess the cross classifying specificities among the kinase-specific models containing the similar substrate site motifs, a particular group is regarded as the positive set and the other groups are regarded as the negative sets one by one. For instance, in the first row the classifying specificity (Sp) of PKA model corresponding to the PKC, PKB and PKG data sets are 51.4%, 27.5% and 38.6%, respectively. This investigation indicates the cross classifying specificities are relatively lower among the kinases PKA, PKC, PKB, and PKG in basophilic group. Similarly, the Sp values marked in blue are relatively lower between the kinases CDK and MAPK in proline-directed group. We observe that the cross classifying specificities corresponding to the kinase-specific models in the same kinase group, such as basophilic, acidophilic, and proline-directed groups, are relatively lower than the specificities corresponding to the kinase-specific models in different groups. To investigate the effect of including structural characteristics for identifying kinase-specific phosphorylation sites with similar substrate motifs, the cross classifying specificities among the kinase-specific models trained with the combination of sequence and structural information are evaluated. As shown in Table S6 (Additional File 1), almost all of the Sp values are increased, especially for the Sp values marked in red, green, and blue. This investigation demonstrates that the consideration of structural information could improve the predictive specificity when identifying the kinase-specific phosphorylation sites with similar sequence motifs.

## Conclusions

The aim of this work is to develop an integrated method for effectively identifying the kinase-specific phosphorylation sites on protein sequences or three-dimensional structures. With the high-throughput mass spectrometry (MS)-based experiment, the desire to comprehensively annotate the catalytic kinases for *in vivo *phosphorylation sites has been highly motivated. Herein, the proposed method could yield a large-scale prediction of over 100 kinase-specific groups which contain reliable accuracy and stable performance. This study has demonstrated that the kinase-specific models trained with the consideration of 3D structural information could perform better than the models trained with only the sequence information, especially improving the cross classifying specificities among the kinase groups containing similar sequence motifs. Additionally, the proposed method was compared with several popular phosphorylation prediction tools, including PredPhospho, GPS 2.0, PPSP, and KinasePhos 2.0. As given in Table 3, the number of kinase groups, sensitivity and specificity of four well-known kinase groups (PKA, PKC, CK2 and SRC) are compared. GPS 2.0 and our method could provide more than 100 kinase-specific groups for phosphorylation sites prediction. In the independent testing performance of PKA, PKC, CK2 and SRC groups, the proposed method is comparable to GPS 2.0 and outperforms other tools.

**Table 3:** **The comparison among PredPhospho, PPSP, GPS 2.0, KiasePhos 2.0, and our method**.

Tools	PredPhospho	GPS 2.0	PPSP	KinasePhos 2.0	Our method
Method	SVM	GPS	BDT	SVM	SVM

Training feature	Sequence	Sequence	Sequence	Sequence	Sequence + **3D structural information**

Material	PhosphoBase + Swiss-Prot	Phospho.ELM	Phospho.ELM	Phospho.ELM + UniProtKB	Phospho.ELM + UniProtKB

No. of kinase groups	4	**> 100**	68	58	**> 100**

Data input	Sequence	Sequence	Sequence	Sequence	Sequence, **PDB ID or structure**

3D structure visualization	-	-	-	-	**JMol**

PKA group	Sn = 70.1% Sp = 86.4%	Sn = 88.2% Sp = 86.6%	Sn = 86.9% Sp = 83.1%	Sn = 86.9% Sp = 85.6%	**Sn = 89.4%** **Sp = 87.7%**

PKC group	Sn = 70.9% Sp = 86.5%	**Sn = 86.2%** Sp = 83.0%	Sn = 82.9% Sp = 85.5%	Sn = 0.84 Sp = 0.86	Sn = 84.3% **Sp = 89.1%**

CK2 group	Sn = 82.0% **Sp = 92.8%**	Sn = 81.4% Sp = 86.4%	Sn = 84.0% Sp = 90.5%	Sn = 86.2% Sp = 86.4%	**Sn = 88.1%** Sp = 90.2%

SRC group	-	Sn = 82.3% Sp = **86.8%**	Sn = 78.0% Sp = 74.6%	**Sn = 86.4%** Sp = 82.2%	**Sn = 86.4%** Sp = 86.2%

The highlights are marked in bold. For PKA group, our method has highest sensitivity and specificity. For PKC group, GPS 2.0 has highest sensitivity and our method has highest specificity. For CK2 group, our method has highest sensitivity and PredPhospho has highest specificity. For SRC group, our method has highest sensitivity and GPS 2.0 has highest specificity.

Abbreviation: SVM, support vector machine; MCL, Markov cluster algorithm; GPS, group-based phosphorylation scoring method; BDT, Bayesian decision theory; MDD, maximal dependence decomposition; HMM, hidden Markov model; AAC, amino acid composition; CP, coupling pattern; SA, structural alphabet; Sn, sensitivity; Sp, specificity; Acc, accuracy.

## Availability

The PhosK3D can be accessed via a web interface, and is freely available to all interested users at http://csb.cse.yzu.edu.tw/PhosK3D/. All of the data set used in this work is also available for download from the website.

## Competing interests

The authors declare that they have no competing interests exist.

## Authors' contributions

TYL conceived and supervised the project. MGS were responsible for the design, computational analyses, implemented the web-based tool, and drafted the manuscript with revisions provided by TYL. All authors read and approved the final manuscript.

## Supplementary Material

Additional File 1 **Supplementary Tables**. Contains additional Tables showing further results in the study Click here for file

## Declarations

The authors sincerely appreciate the National Science Council of the Republic of China for financially supporting this research and publication under Contract Number of NSC 101-2628-E-155-002-MY2.

This article has been published as part of *BMC Bioinformatics *Volume 14 Supplement 16, 2013: Twelfth International Conference on Bioinformatics (InCoB2013): Bioinformatics. The full contents of the supplement are available online at http://www.biomedcentral.com/bmcbioinformatics/supplements/14/S16.
