# Supplementary material for: Incorporating substrate sequence motifs and spatial amino acid composition to identify kinase-specific phosphorylation sites on protein three-dimensional structures
Source: BMC Bioinformatics. 2013 Oct 22;14(Suppl 16):S2. doi: 10.1186/1471-2105-14-S16-S2 (PMC3853090; doi:10.1186/1471-2105-14-S16-S2)
Supplement: Additional File 1 — Supplementary Tables. Contains additional Tables showing further results in the study [file 1471-2105-14-S16-S2-S1.docx]

# SUPPLEMENTARY MATERIALS

**Tables**

**Table S1. Summary table of the previously developed phosphorylation site prediction tools.**

**Table S2. Data statistics and sequence-based characteristics of 122 kinase-specific substrate groups.**

**Table S3. Data statistics and structural characteristics of 21 kinase-specific substrate groups on 3D structures.**

**Table S4. The performances of cross-validation evaluation and independent testing based on sequenced characteristics.**

**Table S1. Summary table of the previously developed phosphorylation site prediction tools.**

| **Tool** | **Reference** | **Material** | **Feature** | **Method** | **Kinase group** | **Proposed predictive performance** | | | |
| --- | --- | --- | --- | --- | --- | --- | --- | --- | --- |
|  |  |  |  |  |  | **Overall** | **PKA** | **PKC** | **CK2** |
| NetPhos | Blom et al., 1999 | PhosphoBase | sequence | ANN | - | Sn=69%~96% | - | - | - |
| Scansite | Obenauer et al., 2003 | Swiss-Prot+TrEMBL+ Genpept+Ensembl | sequence | PSSM (motif-based service) | - | N/A | - | - | - |
| DISPHOS | Lakoucheva et al., 2004 | Swiss-Prot+PhosphoBase | predicted protein disordered region and secondary structure | Logistic regression models | - | Serine Ac=76%  Threonine Ac=81%  Tyrosine Ac=83% | - | - | - |
| rBPNN | Berry et al., 2004 | PhosphoBase | sequence | BPNN, decision tree , rBBFNN | - | BPNN: Ac=89.65±1.64, rBBFNN:Ac=87.77±1.05  C4.5: Ac=90.43±2.03 | - | - | - |
| AutoMotif | Plewczynski et al., 2005 | Swiss-Prot (12 types of PTM) | sequence | SVM | - | Precision > 70% (12 types of PTM) | Sn=41% Pre=75% | Sn=17% Pre=83% | Sn=11% Pre=53% |
| PredPhospho | Jong Hun Kim et al., 2004 | Swiss-Prot+PhosphoBase | sequence | SVM | 4 | Ac = 76 - 91% | Ac=89.98% Sn=88.32% Sp=91.11% | Ac=82.9% Sn=78.71% Sp=85.79% | Ac=91.47% Sn=83.9% Sp=96.43% |
| NetPhosK | Blom et al., 2004 | Swiss-Prot, PhosphoBase, PhosphoSite | sequence | ANN | 17 | Sn = 84%  Sp = 76% | - | - | - |
| GPS | Feng-Feng Zhou et al., 2004 | PhosphoBase, Phospho.ELM | sequence | Clustering or Segmentation | 71 | Sn = 94.44%  Sp = 97.14% | - | - | - |
| KinasePhos | Huang et al., 2005 | PhosphoBase, Swiss-Prot | sequence | MDD + HMM | 18 | Serine Ac = 86%  Threonine Ac = 91%  Tyrosine Ac = 84% | Sn = 0.91  Sp = 0.86 | Sn = 0.80  Sp = 0.87 | Sn = 0.87  Sp = 0.85 |
| Li et al. | Li et al., 2005 | PhosphoBase | sequence | kNN measured by Manhattan distance | - | - | Sn=~87.36% Sp=~99.07% | - | Sn=~67.88% Sp=~99.16% |
| PPSP | Yu Xue et al., 2006 March | Phospho.ELM | sequence | BDT | 68 | N/A | Sn=88.88% Sp=90.57% | N/A | Sn=82.99% Sp=87.59% |
| pkaPS | Neuberger et al.,2007 January | UniProt+Phospho.ELM | sequence | simplified kinase-substrate binding model | - | N/A | Sn=~96% Sp=~94% | N/A | N/A |
| KinasePhos 2.0 | Wong, Lee et al., 2007 | Swiss-Prot+Phospho.ELM | Sequence + coupling pattern | SVM | 58 | Serine Ac = 90%  Threonine Ac = 93%  Tyrosine Ac = 88% | Sn = 0.92  Sp = 0.89 | Sn = 0.84  Sp = 0.86 | Sn = 0.87  Sp = 0.86 |
| GANNPhos | Tang et al., 2007 | Phospho.ELM | sequence | GA+NN | - | S: Ac=81.3~81.8%, Sn=80.5~80.9%, Sp=82.7~83.5% T: Ac=77.5~81.2% , Sn=74.3~77.6%, Sp=83.1~86.4% Y: Ac=74~80.2% , Sn=72.5~76.6%, Sp=77.3~85.6% | N/A | N/A | N/A |
| AutoMotif 2.0 | Plewczynski et al., 2007 | UniProt(06.2007)+Swiss-Prot | sequence | SVM | - | Precision > 90% | Sn=14% Precision=86% | Sn=5% Precision=100% | Sn=6% Precision=80% |
| PHOSIDA | Gnad, Ren et al. 2007 | PHOSIDA | Sequence+ASA+  evolutionary conservation | SVM | - | Serine Ac = 91.75%  Threonine Ac = 81%  Tyrosine Ac = 76.2% | N/A | N/A | N/A |
| MetaPredPS | Ji wan et al.,2008 | Swiss-Prot+PhosphoSite+ Phospho.ELM | - | voting from GPS, KinasePho, NetPhosK, PPSP, PredPhospho, Scansite | - | N/A | Sn=88.3% Sp=82.8% Ac=85% | Sn=77.3% Sp=79.1% Ac=78.4% | Sn=87.8% Sp=90.4% Ac=89.3% |
| NetPhorest | Miller et al., 2008 | Phospho.ELM | Sequence | Linear motif atlas | 179 kinases | N/A | N/A | N/A | N/A |
| CRPhos | Dang TH et al,2008 | Phospho.ELM | Sequence | Conditional random field | N/A | N/A | N/A | N/A | N/A |
| PhoScan | Li et al.,2008 | Swiss-Prot | Sequence | Log-odds ratio | N/A | Sn = 90%  Sp = 90% | N/A | N/A | N/A |
| GPS2.0 | Xue,Y.,et al., 2008 | Phospho.ELM | Sequence | Hierarchy | 408 kinases | Ac = 92.19% | Sn =83.09%  Sp=95.04% | N/A | N/A |
| PostMod | Inkyung Jung et al., 2010 | Phospho.ELM | Sequence+ evolutionary information | noise-reducing algorithm | 48 | Ac =0.93  Pre =0.67  Recall =0.40 | Ac=0.95 | Ac=0.93 | Ac=0.95 |
| Musite | Gao et al., 2010 | UniProtKB, Phospho.ELM, PhosphoPep, PhosPhAt | KNN + disorder score + amino acid frequency | SVM | 13 | N/A | Sn=85.47%  Sp=90.0% | N/A | Sn=83.63%  Sp=90.0% |
| PlantPhos | Lee et al., 2011 | PhosPhAt | Sequence + MDD | SVM | N/A | Serine Ac = 82.4%  Threonine Ac = 78.6%  Tyrosine Ac = 89.0% | N/A | N/A | N/A |
| PhosphoRice | Que et al., 2012 | Swiss-Prot | Sequence | voting from NetPhos 2.0, KinasePhos, NetPhosK, Disphos, PredPhospho, Scansite | N/A | Ac = 73.8% | N/A | N/A | N/A |

Abbreviation: ANN, artificial neural networks; BPNN, back propagation neural network; PSSM, position-specific scoring matrix; SVM, support vector machine; MDD, maximal dependency decomposition; HMM, hidden Markov model; KNN, k-Nearest Neighbor; BDT, Bayesian decision theory; GA, genetic algorithm; ASA, accessible surface area; Ac, accuracy; Sn, sensitivity; Sp, specificity; Pre, precision

**Table S2. Data statistics and sequence-based characteristics of 122 kinase-specific substrate groups.**

| **#** | **Group** | **Kinase family** | **Kinase member** | **Number of substrate sites** | **Entropy plot of sequence logo** | **Computed solvent accessibility** | **Computed secondary structure** |
| --- | --- | --- | --- | --- | --- | --- | --- |
|  |  |  |  |  |  |  |  |
| 1 | AGC | PKB | AKT1, AKT2, PKB | 197 | 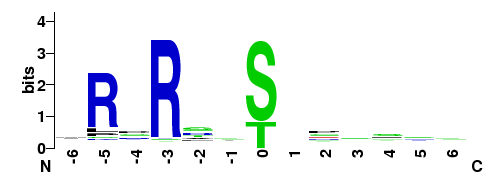 | 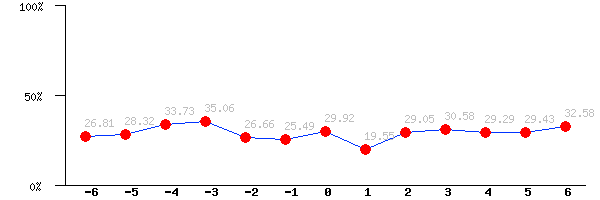 | 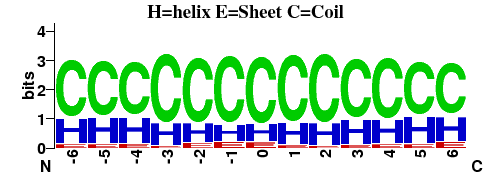 |
| 2 | AGC | ROCK | ROCK1, ROCK2 | 97 | 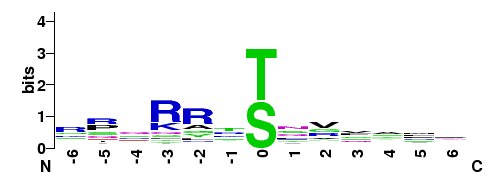 | 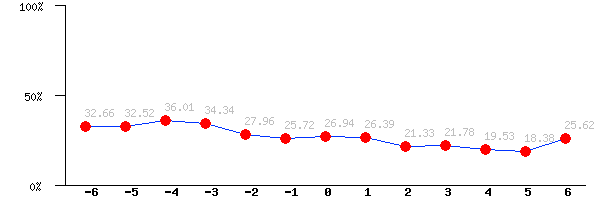 | 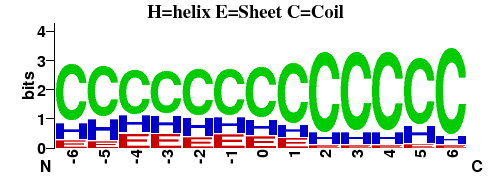 |
| 3 | AGC | GEK | DMPK, MRCKa | 19 | 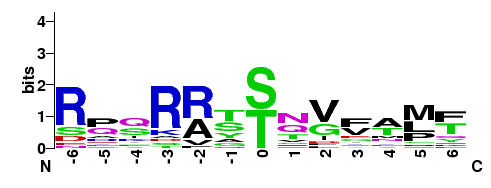 | 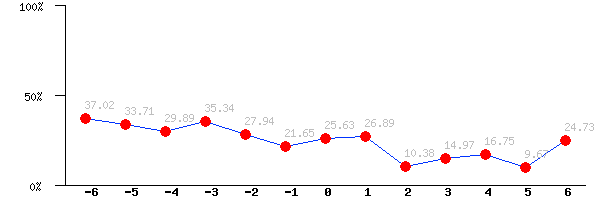 | 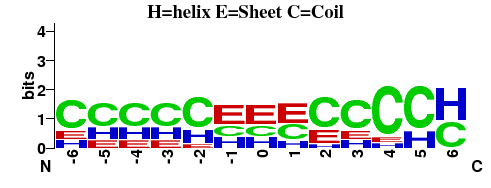 |
| 4 | AGC | GRK | GPRK4, GPRK5, GPRK6, GPRK7, RHOK | 82 | 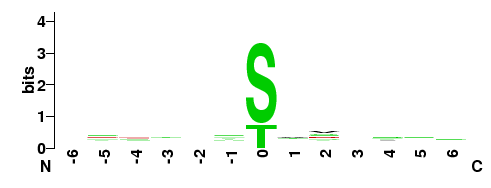 | 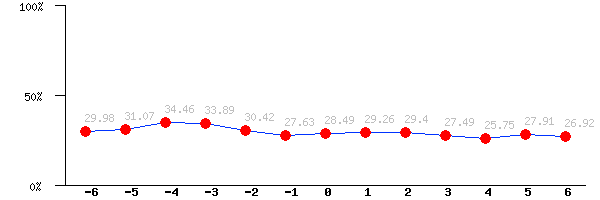 | 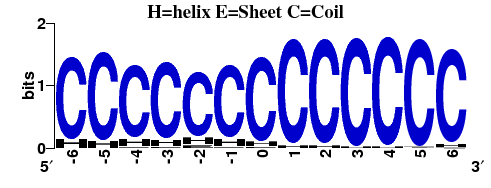 |
| 5 | AGC | BARK | BARK1, BARK2 | 39 | 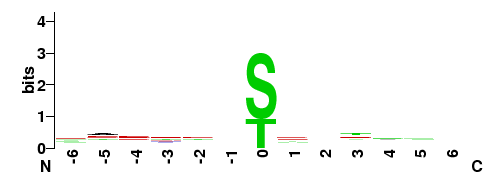 | 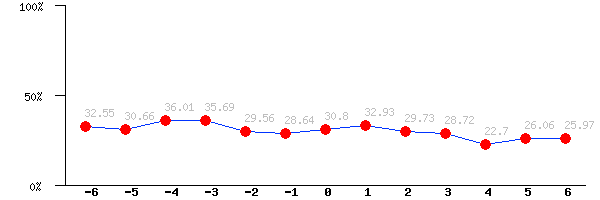 | 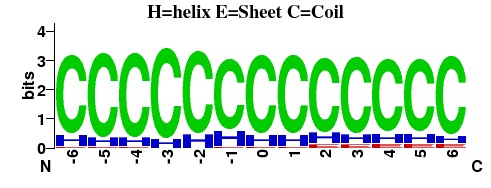 |
| 6 | AGC | NDR | LATS1, LATS2, NDR1, NDR2 | 16 | 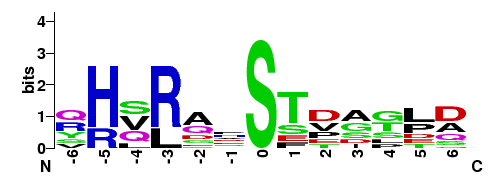 | 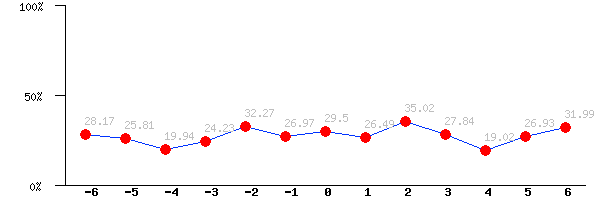 | 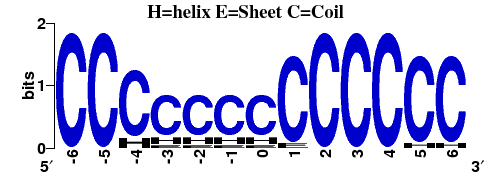 |
| 7 | AGC | PKA | PKACa, PKACb, PKACg | 546 | 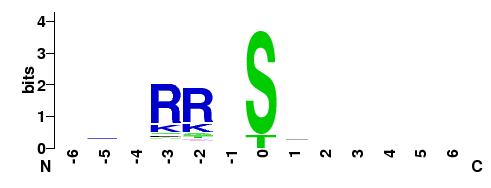 | 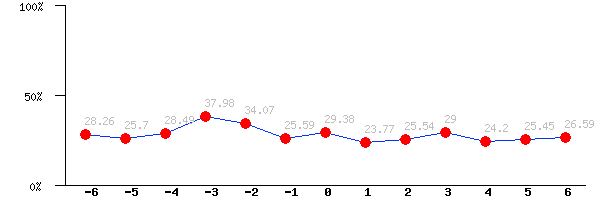 | 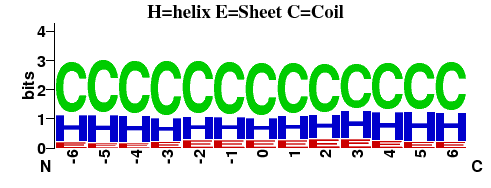 |
| 8 | AGC | PDK1 | PDK1 | 77 | 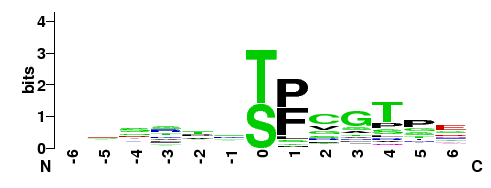 | 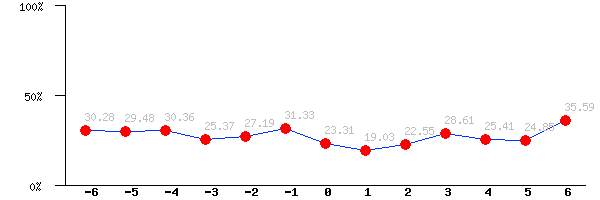 | 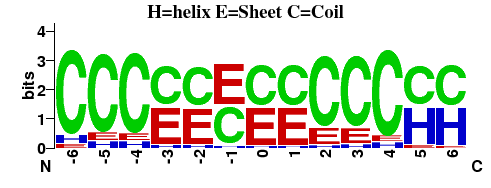 |
| 9 | AGC | PKC | PKC PKCa PKCb PKCd PKCg PKCt PKCe PKCh PKCi PKCz | 652 | 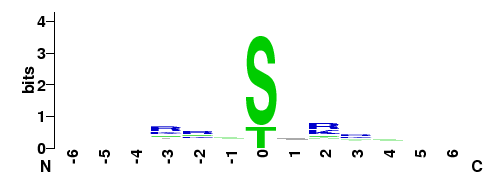 | 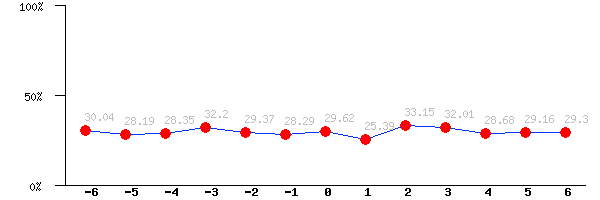 | 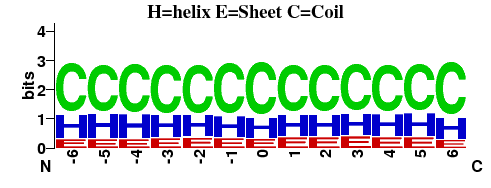 |
| 10 | AGC | PKC Alpha | PKCa PKCb PKCd PKCg | 207 | 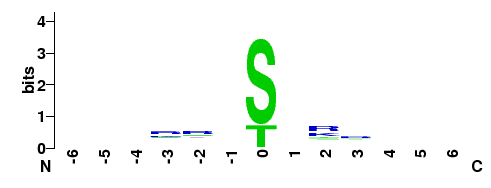 | 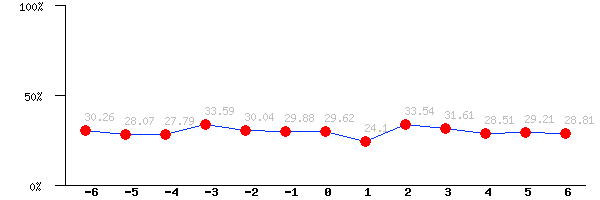 | 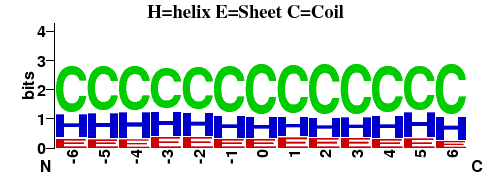 |
| 11 | AGC | PKC Delta | PKCd PKCt | 42 | 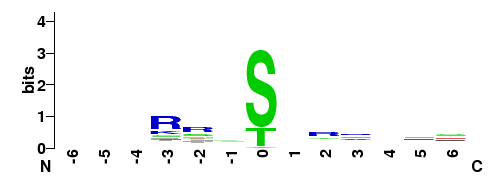 | 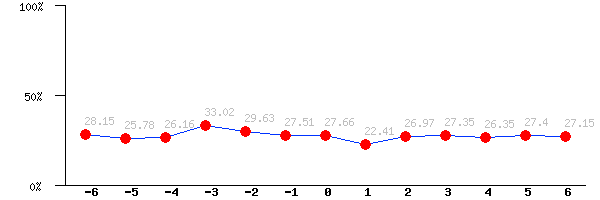 | 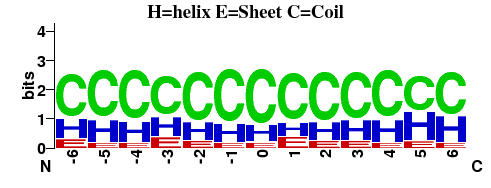 |
| 12 | AGC | PKC Eta | PKCe PKCh | 30 | 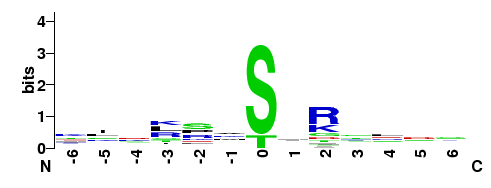 | 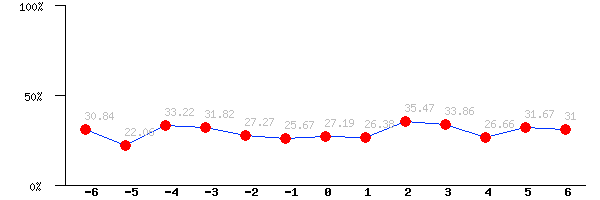 | 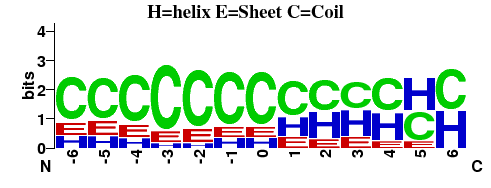 |
| 13 | AGC | PKC Iota | PKCi PKCz | 28 | 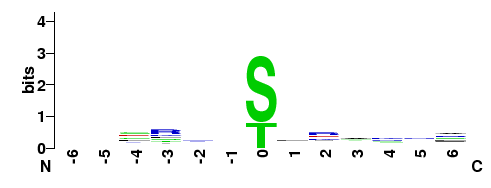 | 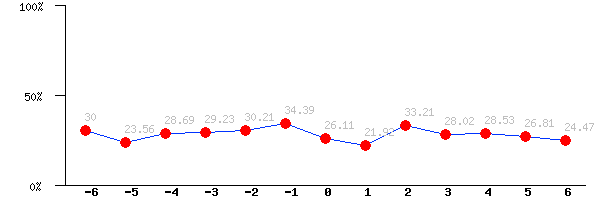 | 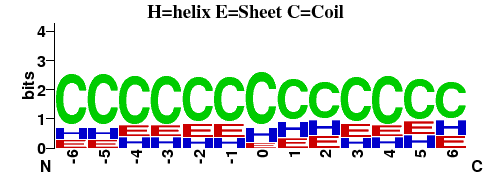 |
| 14 | AGC | PKG | PKG PKG1 PKG2 | 64 | 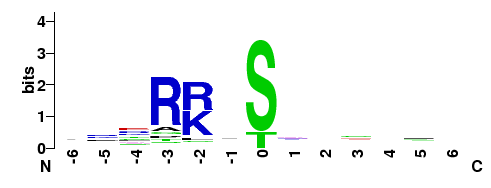 | 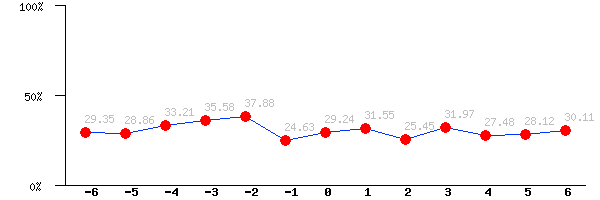 | 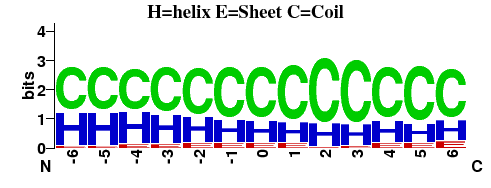 |
| 15 | AGC | PKN | PKN1 PKN2 PKNB PKND PKNE PKNF PKNK | 33 | 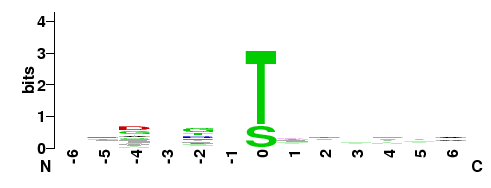 | 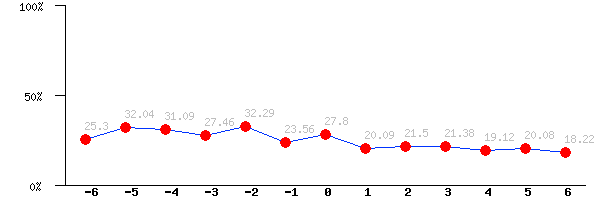 | 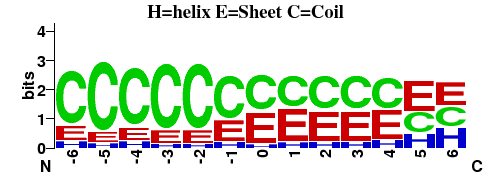 |
| 16 | AGC | RSK | RSK RSK1 RSK2 RSK3 | 106 | 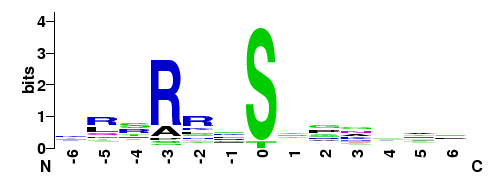 | 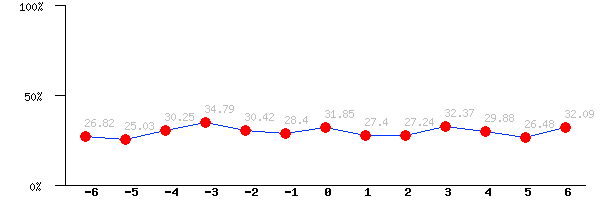 | 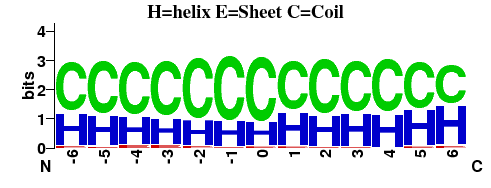 |
| 17 | AGC | p70 | p70S6K p70S6Kb | 25 | 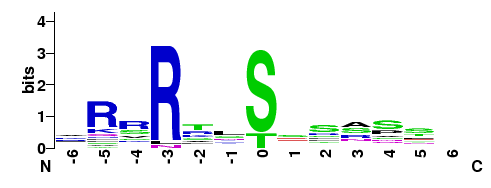 | 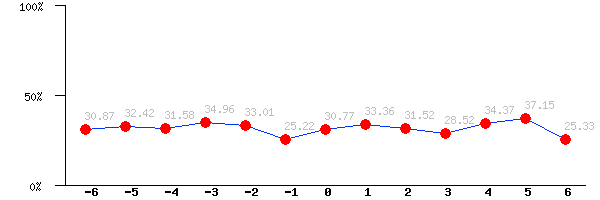 | 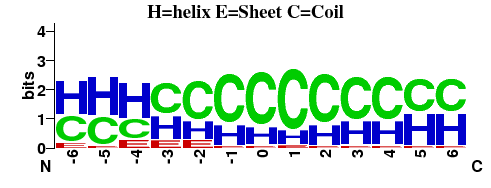 |
| 18 | AGC | MSK | MSK1 MSK2 | 68 | 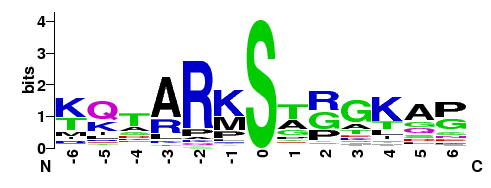 | 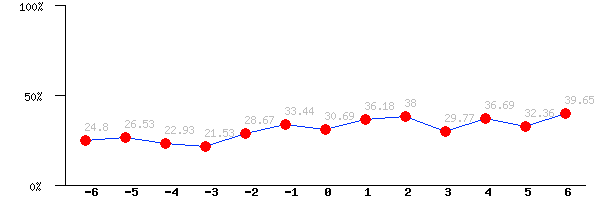 | 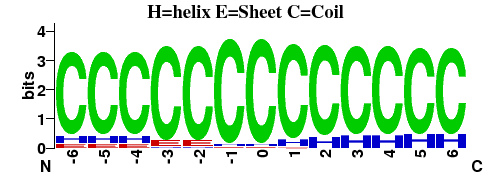 |
| 19 | AGC | SGK | SGK SGK1 SGK3 | 69 | 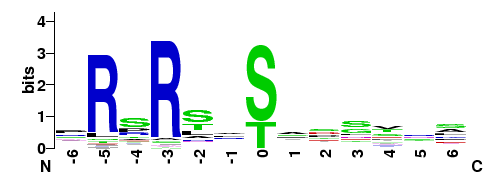 | 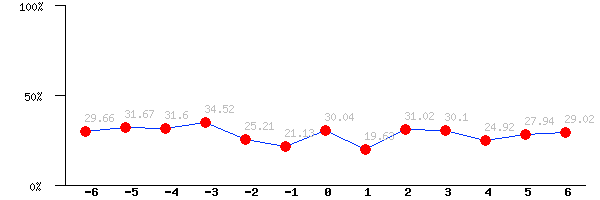 | 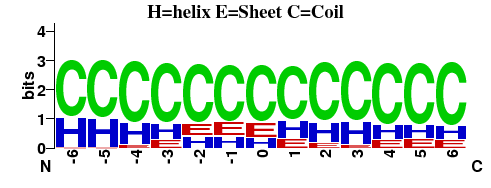 |
| 20 | Atypical | ChaK | ChaK1 ChaK2 | 8 | 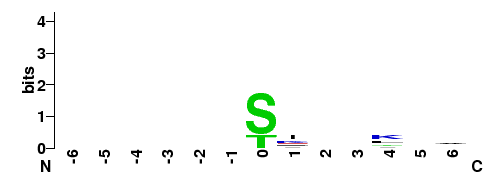 | 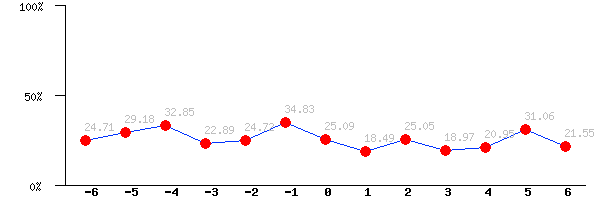 | 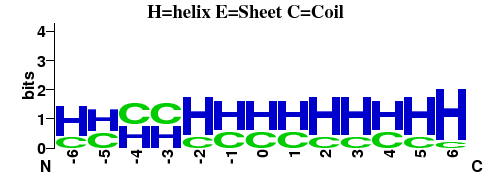 |
| 21 | Atypical | PDHK | PDHK1 PDHK2 PDHK3 PDHK4 BCKDK | 21 | 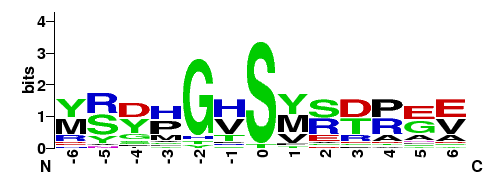 | 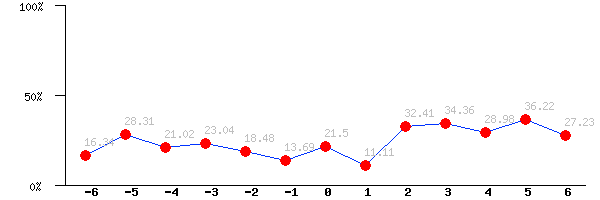 | 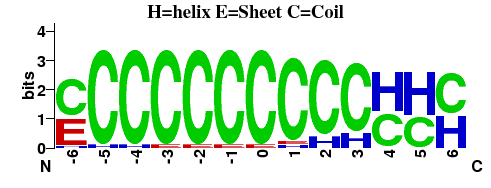 |
| 22 | Atypical | ATM | ATM ATR | 181 | 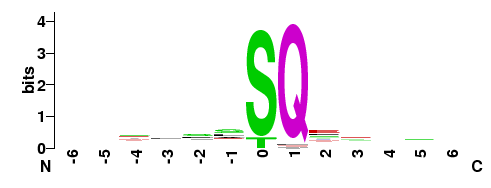 | 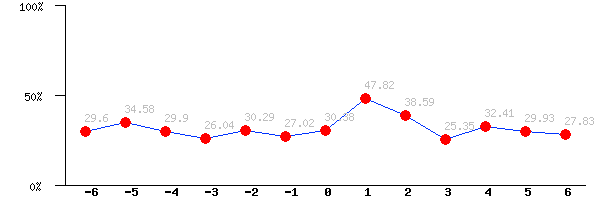 | 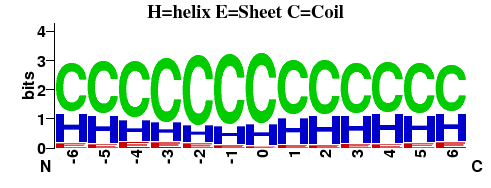 |
| 23 | Atypical | DNAPK | DNAPK | 35 | 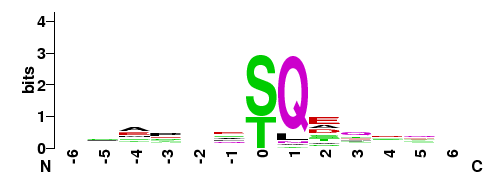 | 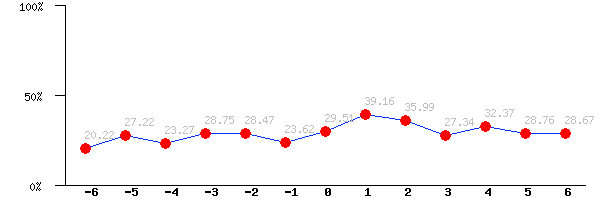 | 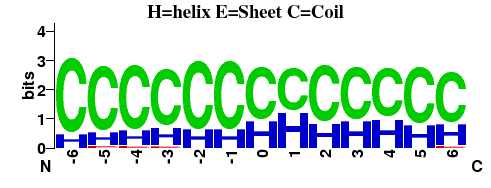 |
| 24 | Atypical | FRAP | FRAP | 31 | 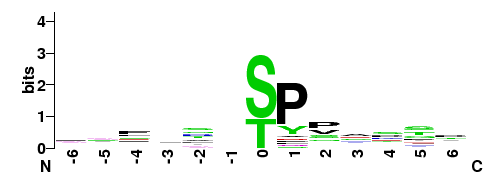 | 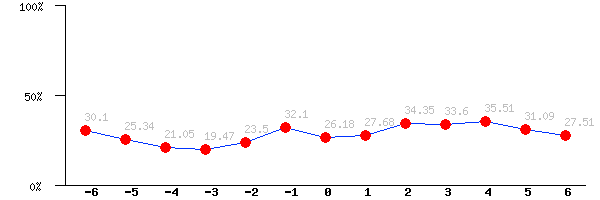 | 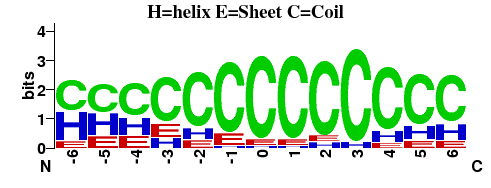 |
| 25 | Atypical | TAF1 | TAF1 | 8 | 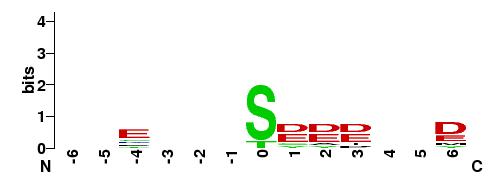 | 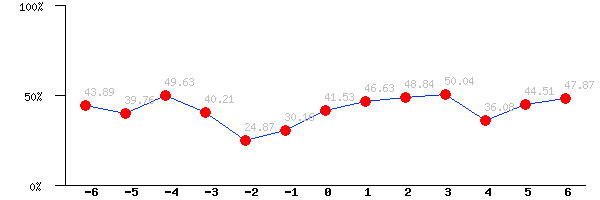 | 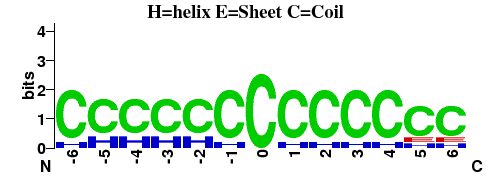 |
| 26 | CAMK | CAMK1 | CaMK1 CaMK1a CaMK1d CaMK4 CaMK | 51 | 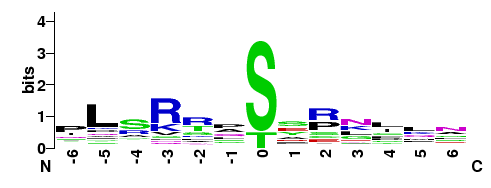 | 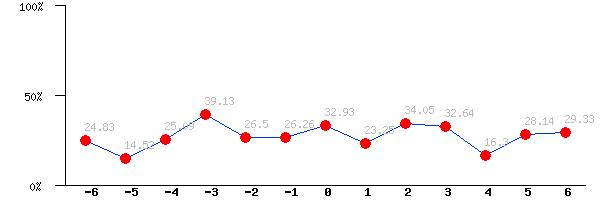 | 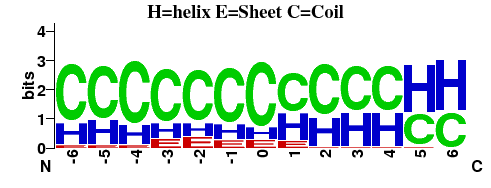 |
| 27 | CAMK | CAMK2 | CaMK2 CaMK2a CaMK2b CaMK2d CaMK2g CaMK | 148 | 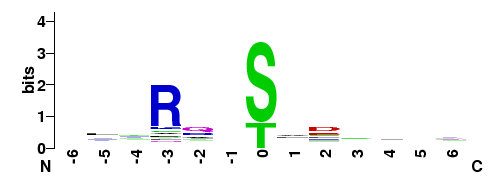 | 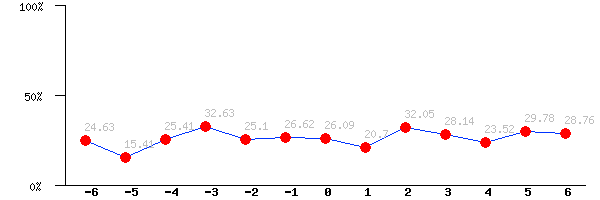 | 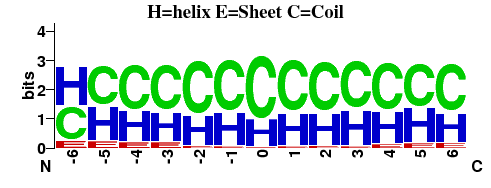 |
| 28 | CAMK | NuaK | NuaK1 NuaK2 | 9 | 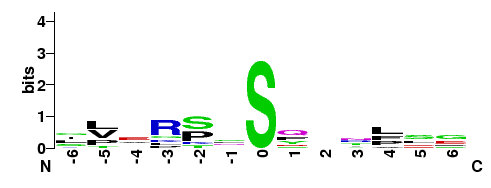 | 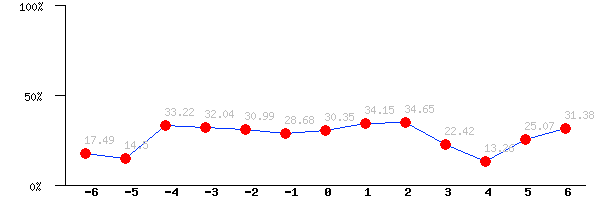 | 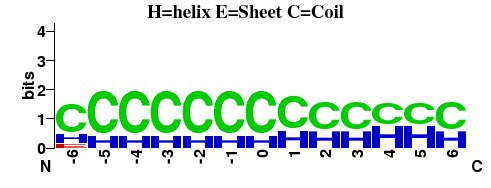 |
| 29 | CAMK | QIK | QIK SIK | 24 | 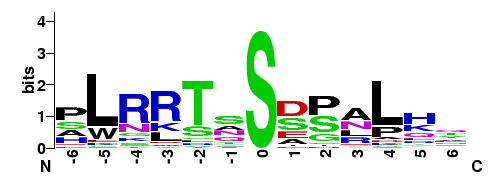 | 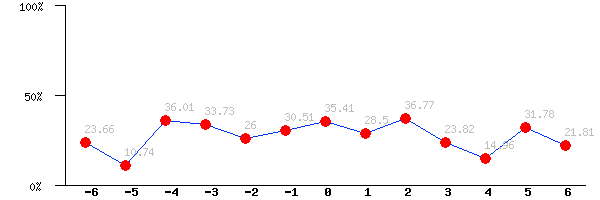 | 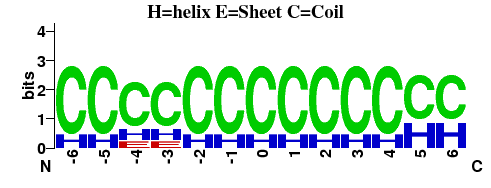 |
| 30 | CAMK | MELK | MELK | 21 | 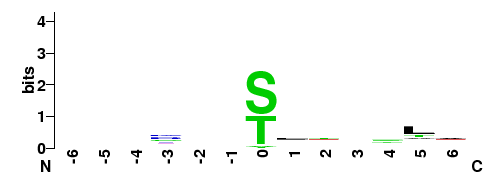 | 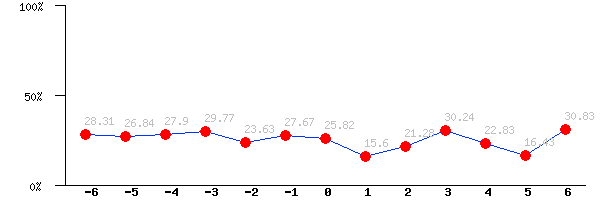 | 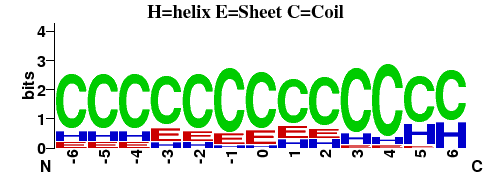 |
| 31 | CAMK | AMPK | AMPKa1, AMPKa2 | 88 | 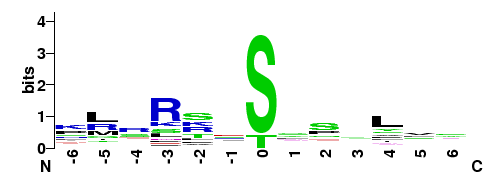 | 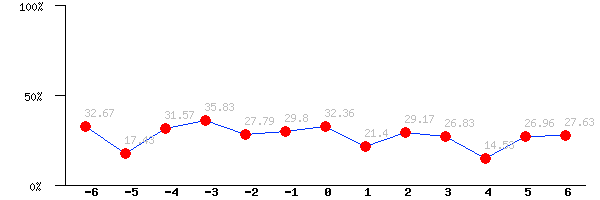 | 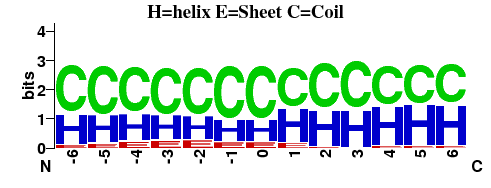 |
| 32 | CAMK | CHK1 | CHK1 | 23 | 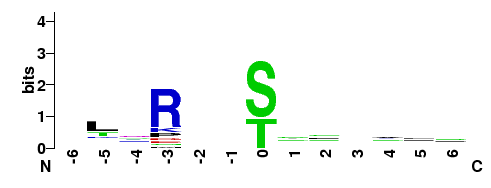 | 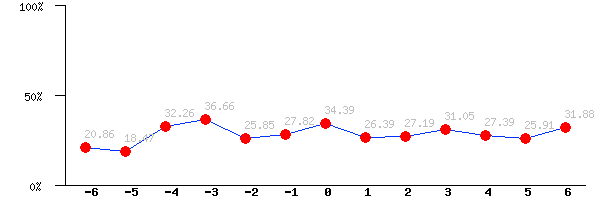 | 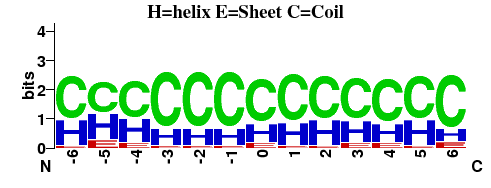 |
| 33 | CAMK | CHK2 | CHK2 | 35 | 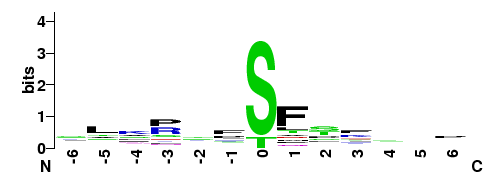 | 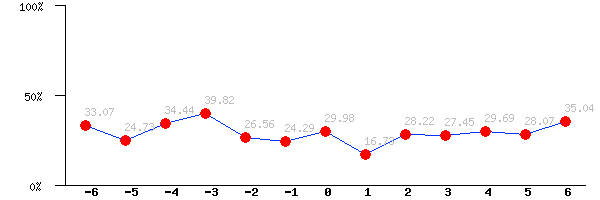 | 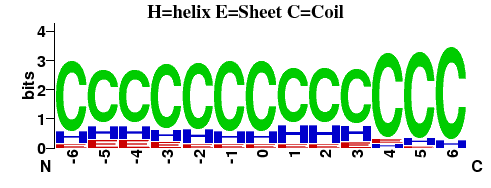 |
| 34 | CAMK | LKB | LKB1 | 35 | 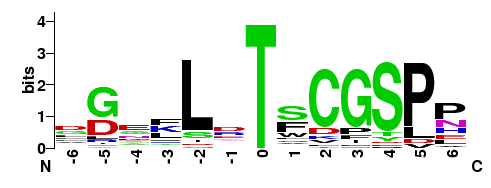 |  |  |
| 35 | CAMK | MARK | MARK MARK1 MARK2 MARK3 | 24 |  |  |  |
| 36 | CAMK | BRSK | BRSK1 BRSK2 | 10 |  |  |  |
| 37 | CAMK | DAPK | DAPK DAPK1 DAPK2 DAPK3 | 64 |  |  |  |
| 38 | CAMK | MAPKAPK | MAPKAPK2 MAPKAPK3 MAPKAPK5 | 68 |  |  |  |
| 39 | CAMK | MNK | MNK1 MNK2 | 11 |  |  |  |
| 40 | CAMK | MLCK | MLCK TTN | 21 |  |  |  |
| 41 | CAMK | PHK | PHK | 28 |  |  |  |
| 42 | CAMK | PIM | PIM1 PIM2 PIM3 | 19 |  |  |  |
| 43 | CAMK | PKD | PKD1 PKD2 PKD3 | 32 |  |  |  |
| 44 | CK1 | CK1 | CK1 CK1a CK1d CK1e | 267 |  |  |  |
| 45 | CK1 | TTBK | TTBK1 | 10 |  |  |  |
| 46 | CK1 | VRK | VRK1 VRK2 | 16 |  |  |  |
| 47 | CMGC | CDK | CDK10 CDK11 CDK4 CDK5 CDK6 CDK8 CDK9 | 193 |  |  |  |
| 48 | CMGC | CDK7 | CDK7 | 21 |  |  |  |
| 49 | CMGC | CDC2 | CDC2, CDK2, CDK3 | 436 |  |  |  |
| 50 | CMGC | CK2 | CK2, CK2a, CK2b | 488 |  |  |  |
| 51 | CMGC | CLK | CLK1, CLK2, CLK3, CLK4 | 12 |  |  |  |
| 52 | CMGC | HIPK | HIPK1 HIPK2 HIPK3 HIPK4 | 33 |  |  |  |
| 53 | CMGC | DYRK | DYRK1A, DYRK1B, DYRK2, DYRK3 | 37 |  |  |  |
| 54 | CMGC | GSK | GSK3A, GSK3B | 148 |  |  |  |
| 55 | CMGC | MAPK | JNK1(MAPK8), JNK2(MAPK9), JNK3(MAPK10), p38a(MAPK14), p38b(MAPK11), p38d(MAPK13), p38g(MAPK12), Erk1(MAPK3), Erk2(MAPK1), Erk3(MAPK6), Erk4(MAPK4), Erk5(MAPK7), Erk7(MAPK15), NLK | 663 |  |  |  |
| 56 | CMGC | JNK | JNK1(MAPK8), JNK2(MAPK9), JNK3(MAPK10) | 111 |  |  |  |
| 57 | CMGC | p38 | p38a(MAPK14), p38b(MAPK11), p38d(MAPK13), p38g(MAPK12) | 106 |  |  |  |
| 58 | CMGC | ERK | Erk1(MAPK3), Erk2(MAPK1), Erk3(MAPK6), Erk4(MAPK4), Erk5(MAPK7), Erk7(MAPK15) | 357 |  |  |  |
| 59 | CMGC | nmo | NLK | 16 |  |  |  |
| 60 | Other | Aur | AurB AurC AurA | 118 |  |  |  |
| 61 | Other | AurB | AurB | 59 |  |  |  |
| 62 | Other | AurA | AurA | 37 |  |  |  |
| 63 | Other | CAMKK | CaMKK1 CaMKK2 | 17 |  |  |  |
| 64 | Other | IKK | IKKa IKKb IKKe TBK1 | 86 |  |  |  |
| 65 | Other | NEK | NEK11 NEK2 NEK6 NEK7 NEK9 | 33 |  |  |  |
| 66 | Other | PKR | PKR | 13 |  |  |  |
| 67 | Other | PLK | PLK1 PLK2 PLK3 PLK4 | 118 |  |  |  |
| 68 | Other | TTK | TTK | 13 |  |  |  |
| 69 | Other | ULK | ULK1 ULK3 | 10 |  |  |  |
| 70 | Other | WEE | MYT1 Wee1 Wee1B | 14 |  |  |  |
| 71 | Other | WNK | Wnk1, Wnk4 | 14 |  |  |  |
| 72 | STE | MAP3K | MAP3K1, MAP3K2, MAP3K3, MAP3K4, MAP3K5, MAP3K6, MAP3K7, MAP3K8 | 31 |  |  |  |
| 73 | STE | PAK | PAK6 PAK1 PAK4 PAK5 PAK2 PAK3 | 116 |  |  |  |
| 74 | STE | PAKB | PAK4, PAK5, PAK6 | 14 |  |  |  |
| 75 | STE | PAKA | PAK1, PAK2, PAK3 | 90 |  |  |  |
| 76 | STE | YSK | MST3, MST4, YSK1 | 6 |  |  |  |
| 77 | STE | KHS | GCK HPK1 | 7 |  |  |  |
| 78 | STE | MSN | HGK, MINK, NRK, TNIK | 9 |  |  |  |
| 79 | STE | MST | MST1, MST2 | 55 |  |  |  |
| 80 | STE | MAP2K | MAP2K5, MAP2K1, MAP2K2, MAP2K3, MAP2K4, MAP2K6, MAP2K7 | 101 |  |  |  |
| 81 | TK | Abl | ABL1(Abl), ABL2(ARG) | 121 |  |  |  |
| 82 | TK | Ack | ACK | 10 |  |  |  |
| 83 | TK | Axl | AXL, MER, MERTK | 10 |  |  |  |
| 84 | TK | Csk | CSK, CTK | 29 |  |  |  |
| 85 | TK | DDR | DDR1, DDR2 | 20 |  |  |  |
| 86 | TK | EGFR | EGFR, ErbB2, ErbB3, ErbB4 | 79 |  |  |  |
| 87 | TK | Eph | EphA2, EphA3, EphA4, EphA8, EphB1, EphB2, EphB3, EphB5 | 32 |  |  |  |
| 88 | TK | FAK | FAK PYK2 | 28 |  |  |  |
| 89 | TK | Fer | FER | 14 |  |  |  |
| 90 | TK | Fes | FES | 11 |  |  |  |
| 91 | TK | FGFR | FGFR1, FGFR2, FGFR3, FGFR4 | 43 |  |  |  |
| 92 | TK | InsR | INSR | 51 |  |  |  |
| 93 | TK | IGF1R | IGF1R | 26 |  |  |  |
| 94 | TK | JakA | JAK1, JAK2, JAK3 | 59 |  |  |  |
| 95 | TK | TYK2 | TYK2 | 20 |  |  |  |
| 96 | TK | Met | MET, RON | 31 |  |  |  |
| 97 | TK | PDGFR | PDGFRa, PDGFRb | 43 |  |  |  |
| 98 | TK | Fms | FMS | 15 |  |  |  |
| 99 | TK | Kit | KIT | 8 |  |  |  |
| 100 | TK | Ret | RET | 18 |  |  |  |
| 101 | TK | Brk | BRK | 22 |  |  |  |
| 102 | TK | Fgr | FGR | 9 |  |  |  |
| 103 | TK | Fyn | FYN | 66 |  |  |  |
| 104 | TK | HCK | HCK | 18 |  |  |  |
| 105 | TK | Lck | LCK | 64 |  |  |  |
| 106 | TK | LYN | LYN | 73 |  |  |  |
| 107 | TK | Src | SRC | 221 |  |  |  |
| 108 | TK | YES | YES | 6 |  |  |  |
| 109 | TK | SYK | SYK | 61 |  |  |  |
| 110 | TK | ZAP70 | ZAP70 | 21 |  |  |  |
| 111 | TK | Tec | BMX, ITK, TEC, TXK, BTK | 47 |  |  |  |
| 112 | TK | BTK | BTK | 19 |  |  |  |
| 113 | TK | ITK | ITK | 9 |  |  |  |
| 114 | TK | TXK | TXK | 8 |  |  |  |
| 115 | TK | Tie | TIE1, TIE2 | 9 |  |  |  |
| 116 | TK | Trk | TRKA TRKB | 17 |  |  |  |
| 117 | TK | VEGFR | FLT1 FLT3 FLT4 KDR | 36 |  |  |  |
| 118 | TKL | IRAK | IRAK1, IRAK4 | 9 |  |  |  |
| 119 | TKL | RAF | BRAF, RAF1 | 9 |  |  |  |
| 120 | TKL | STKR Type1 | ALK4 BMPR1B TGFbR1 | 17 |  |  |  |
| 121 | TKL | STKR Type2 | TGFbR2 | 11 |  |  |  |
| 122 | TKL | TGFbR | TGFbR1, TGFbR2 | 20 |  |  |  |

**Table S3. Data statistics and structural characteristics of 21 kinase-specific substrate groups on 3D structures.**

| **Kinase group** | **Number of substrate sites** | **Sequenced AAC** | **Spatial AAC (distance≦10**Å**)** | **Structural alphabet** | **Solvent accessibility by DSSP (%)** | **Secondary structure by DSSP** |
| --- | --- | --- | --- | --- | --- | --- |
| **Phosphorylated Serine (pSer)** | | | | | | |
| CDK | 11 |  |  |  |  |  |
| CK1 | 10 |  |  |  |  |  |
| CK2 | 24 |  |  |  |  |  |
| MAPK | 17 |  |  |  |  |  |
| PIKK | 15 |  |  |  |  |  |
| PKA | 56 |  |  |  |  |  |
| PKB | 12 |  |  |  |  |  |
| PKC | 50 |  |  |  |  |  |
| PKG | 10 |  |  |  |  |  |
| PLK | 10 |  |  |  |  |  |
| STE20 | 10 |  |  |  |  |  |
| **Phosphorylated Threonine (pThr)** | | | | | | |
| MAPK | 13 |  |  |  |  |  |
| PKA | 10 |  |  |  |  |  |
| PKC | 13 |  |  |  |  |  |
| STE20 | 10 |  |  |  |  |  |
| **Phosphorylated Tyrosine (pTyr)** | | | | | | |
| Abl | 18 |  |  |  |  |  |
| EGFR | 10 |  |  |  |  |  |
| FGFR | 10 |  |  |  |  |  |
| InsR | 15 |  |  |  |  |  |
| Src | 57 |  |  |  |  |  |
| Syk | 11 |  |  |  |  |  |

Abbreviation: AAC, amino acid composition.

**Table S4. The performances of cross-validation evaluation and independent testing based on sequenced characteristics.**

| **#** | **Group** | **Kinase family** | **Entropy plot of sequence logo** | **Cross-validation evaluation^a^** | | | | | **Independent testing^b^** | | | | |
| --- | --- | --- | --- | --- | --- | --- | --- | --- | --- | --- | --- | --- | --- |
|  |  |  |  | **Number of positive data** | **Number of negative data** | **Sn** | **Sp** | **Acc** | **Number of positive data** | **Number of negative data** | **Sn** | **Sp** | **Acc** |
| 1 | AGC | PKB |  | 197 | 197 | 98.1% | 94.1% | 96.1% | 54 | 108 | 96.3% | 97.2% | 96.9% |
| 2 | AGC | ROCK |  | 97 | 97 | 90.9% | 89.1% | 90.0% | 23 | 46 | 73.9% | 84.8% | 81.2% |
| 3 | AGC | GEK |  | 19 | 19 | 94.7% | 97.4% | 96.0% | 2 | 4 | 100% | 100% | 100% |
| 4 | AGC | GRK |  | 82 | 82 | 81.6% | 79.5% | 80.6% | 10 | 20 | 80.0% | 75.0% | 76.7% |
| 5 | AGC | BARK |  | 39 | 39 | 80.7% | 83.1% | 81.9% | 11 | 22 | 72.7% | 77.3% | 75.7% |
| 6 | AGC | NDR |  | 16 | 16 | 93.8% | 93.8% | 93.8% | 2 | 4 | 100% | 100% | 100% |
| 7 | AGC | PKA |  | 546 | 546 | 89.2% | 89.4% | 89.3% | 192 | 384 | 90.1% | 88.2% | 88.8% |
| 8 | AGC | PDK1 |  | 77 | 77 | 83.9% | 85.1% | 84.5% | 16 | 32 | 87.5% | 81.3% | 83.3% |
| 9 | AGC | PKC |  | 652 | 652 | 85.9% | 87.2% | 86.6% | 325 | 650 | 84.3% | 89.1% | 87.5% |
| 10 | AGC | PKC Alpha |  | 207 | 207 | 87.2% | 84.8% | 86.0% | 236 | 472 | 84.3% | 90.9% | 88.7% |
| 11 | AGC | PKC Delta |  | 42 | 42 | 89.9% | 90.2% | 90.1% | 61 | 122 | 85.2% | 78.7% | 80.9% |
| 12 | AGC | PKC Eta |  | 30 | 30 | 82.9% | 92.0% | 87.5% | 14 | 28 | 78.6% | 92.9% | 88.1% |
| 13 | AGC | PKC Iota |  | 28 | 28 | 85.9% | 87.2% | 86.6% | 14 | 28 | 78.6% | 85.7% | 83.3% |
| 14 | AGC | PKG |  | 64 | 64 | 89.2% | 91.1% | 90.1% | 20 | 40 | 90.0% | 87.5% | 88.3% |
| 15 | AGC | PKN |  | 33 | 33 | 81.2% | 79.4% | 80.3% | 18 | 36 | 77.8% | 77.8% | 77.8% |
| 16 | AGC | RSK |  | 106 | 106 | 90.5% | 93.1% | 91.8% | 44 | 88 | 81.8% | 88.6% | 86.4% |
| 17 | AGC | p70 |  | 25 | 25 | 92.0% | 95.6% | 93.8% | 2 | 4 | 100% | 100% | 100% |
| 18 | AGC | MSK |  | 68 | 68 | 91.2% | 91.2% | 91.2% | 17 | 34 | 88.2% | 91.2% | 90.2% |
| 19 | AGC | SGK |  | 69 | 69 | 89.2% | 89.4% | 89.3% | 9 | 18 | 100% | 88.9% | 92.6% |
| 20 | Atypical | ChaK |  | 8 | 8 | 86.3% | 76.3% | 81.3% | N/A | N/A | N/A | N/A | N/A |
| 21 | Atypical | PDHK |  | 21 | 21 | 90.3% | 92.4% | 91.4% | 1 | 2 | 100% | 100% | 100% |
| 22 | Atypical | ATM |  | 181 | 181 | 99.1% | 96.0% | 97.6% | 38 | 76 | 92.1% | 100.0% | 97.4% |
| 23 | Atypical | DNAPK |  | 35 | 35 | 98.3% | 98.9% | 98.6% | 12 | 24 | 100% | 95.8% | 97.2% |
| 24 | Atypical | FRAP |  | 31 | 31 | 86.8% | 94.5% | 90.7% | 2 | 4 | 100% | 75.0% | 83.3% |
| 25 | Atypical | TAF1 |  | 8 | 8 | 82.5% | 87.5% | 85.0% | N/A | N/A | N/A | N/A | N/A |
| 26 | CAMK | CAMK1 |  | 51 | 51 | 83.9% | 85.7% | 84.8% | 7 | 14 | 85.7% | 78.6% | 80.9% |
| 27 | CAMK | CAMK2 |  | 148 | 148 | 93.0% | 91.1% | 92.1% | 60 | 120 | 90.0% | 88.3% | 88.9% |
| 28 | CAMK | NuaK |  | 9 | 9 | 86.7% | 86.7% | 86.7% | N/A | N/A | N/A | N/A | N/A |
| 29 | CAMK | QIK |  | 24 | 24 | 89.2% | 89.2% | 89.2% | 5 | 10 | 80.0% | 80.0% | 80.0% |
| 30 | CAMK | MELK |  | 21 | 21 | 90.0% | 84.8% | 87.4% | 1 | 2 | 0% | 100.0% | 66.7% |
| 31 | CAMK | AMPK |  | 88 | 88 | 90.7% | 93.3% | 92.0% | 17 | 34 | 88.2% | 88.2% | 88.2% |
| 32 | CAMK | CHK1 |  | 23 | 23 | 93.4% | 94.0% | 93.7% | 4 | 8 | 100.0% | 100.0% | 100% |
| 33 | CAMK | CHK2 |  | 35 | 35 | 90.2% | 91.1% | 90.6% | 2 | 4 | 50.0% | 100.0% | 83.3% |
| 34 | CAMK | LKB |  | 35 | 35 | 94.3% | 94.3% | 94.3% | 2 | 4 | 100.0% | 100.0% | 100% |
| 35 | CAMK | MARK |  | 24 | 24 | 91.7% | 91.7% | 91.7% | 14 | 28 | 85.7% | 89.3% | 88.1% |
| 36 | CAMK | BRSK |  | 10 | 10 | 90.1% | 89.9% | 90.0% | 1 | 2 | 100.0% | 100.0% | 100% |
| 37 | CAMK | DAPK |  | 64 | 64 | 93.3% | 95.4% | 94.4% | 1 | 2 | 100.0% | 100.0% | 100% |
| 38 | CAMK | MAPKAPK |  | 68 | 68 | 89.6% | 91.9% | 90.8% | 9 | 18 | 77.8% | 88.9% | 85.2% |
| 39 | CAMK | MNK |  | 11 | 11 | 100.0% | 96.3% | 98.2% | 1 | 2 | 100.0% | 50.0% | 66.7% |
| 40 | CAMK | MLCK |  | 21 | 21 | 91.4% | 93.3% | 92.4% | N/A | N/A | N/A | N/A | N/A |
| 41 | CAMK | PHK |  | 28 | 28 | 91.1% | 90.4% | 90.7% | 4 | 8 | 75.0% | 75.0% | 75.0% |
| 42 | CAMK | PIM |  | 19 | 19 | 93.2% | 93.2% | 93.2% | 7 | 14 | 100.0% | 85.7% | 90.5% |
| 43 | CAMK | PKD |  | 32 | 32 | 96.6% | 92.5% | 94.5% | 8 | 16 | 87.5% | 100.0% | 95.8% |
| 44 | CK1 | CK1 |  | 267 | 267 | 92.2% | 90.9% | 91.6% | 139 | 278 | 87.1% | 87.8% | 87.5% |
| 45 | CK1 | TTBK |  | 10 | 10 | 90.0% | 100.0% | 95.0% | 1 | 2 | 100.0% | 100.0% | 100% |
| 46 | CK1 | VRK |  | 16 | 16 | 87.5% | 87.5% | 87.5% | N/A | N/A | N/A | N/A | N/A |
| 47 | CMGC | CDK |  | 193 | 193 | 95.2% | 90.7% | 93.0% | 64 | 128 | 95.2% | 90.7% | 93.0% |
| 48 | CMGC | CDK7 |  | 21 | 21 | 88.4% | 86.7% | 87.6% | 3 | 6 | 100% | 83.3% | 88.9% |
| 49 | CMGC | CDC2 |  | 436 | 436 | 94.3% | 90.5% | 92.4% | 103 | 206 | 96.1% | 88.8% | 91.2% |
| 50 | CMGC | CK2 |  | 488 | 488 | 87.1% | 88.5% | 87.8% | 113 | 226 | 88.5% | 86.3% | 87.0% |
| 51 | CMGC | CLK |  | 12 | 12 | 86.7% | 86.7% | 86.7% | N/A | N/A | N/A | N/A | N/A |
| 52 | CMGC | HIPK |  | 33 | 33 | 92.7% | 88.7% | 90.7% | 3 | 6 | 100% | 83.3% | 88.9% |
| 53 | CMGC | DYRK |  | 37 | 37 | 88.4% | 90.7% | 89.6% | 5 | 10 | 80.0% | 90.0% | 86.7% |
| 54 | CMGC | GSK |  | 148 | 148 | 83.6% | 80.1% | 81.8% | 68 | 136 | 89.7% | 86.0% | 87.2% |
| 55 | CMGC | MAPK |  | 663 | 663 | 91.9% | 91.2% | 91.6% | 220 | 440 | 89.1% | 92.7% | 91.5% |
| 56 | CMGC | JNK |  | 111 | 111 | 92.0% | 92.0% | 92.0% | 50 | 100 | 88.0% | 89.0% | 88.7% |
| 57 | CMGC | p38 |  | 106 | 106 | 90.0% | 94.7% | 92.4% | 48 | 96 | 93.8% | 85.4% | 88.2% |
| 58 | CMGC | ERK |  | 357 | 357 | 92.8% | 91.1% | 92.0% | 122 | 244 | 87.7% | 88.5% | 88.2% |
| 59 | CMGC | nmo |  | 16 | 16 | 100% | 95.6% | 97.8% | N/A | N/A | N/A | N/A | N/A |
| 60 | Other | Aur |  | 118 | 118 | 92.3% | 91.5% | 91.9% | 5 | 10 | 100% | 90% | 93.3% |
| 61 | Other | AurB |  | 59 | 59 | 96.9% | 97.8% | 97.3% | 5 | 10 | 100% | 100% | 100% |
| 62 | Other | AurA |  | 37 | 37 | 94.6% | 93.5% | 94.0% | N/A | N/A | N/A | N/A | N/A |
| 63 | Other | CAMKK |  | 17 | 17 | 100.0% | 94.7% | 97.4% | 1 | 2 | 100% | 100% | 100% |
| 64 | Other | IKK |  | 86 | 86 | 91.0% | 95.8% | 93.4% | 35 | 70 | 82.9% | 84.3% | 83.8% |
| 65 | Other | NEK |  | 33 | 33 | 82.2% | 86.7% | 84.5% | 9 | 18 | 77.8% | 83.3% | 81.5% |
| 66 | Other | PKR |  | 13 | 13 | 83.8% | 94.6% | 89.2% | 16 | 32 | 75.0% | 81.3% | 79.2% |
| 67 | Other | PLK |  | 118 | 118 | 83.1% | 85.6% | 84.4% | 4 | 8 | 75.0% | 75.0% | 75.0% |
| 68 | Other | TTK |  | 13 | 13 | 83.8% | 88.5% | 86.1% | N/A | N/A | N/A | N/A | N/A |
| 69 | Other | ULK |  | 10 | 10 | 90.0% | 100.0% | 95.0% | N/A | N/A | N/A | N/A | N/A |
| 70 | Other | WEE |  | 14 | 14 | 100.0% | 100.0% | 100.0% | 1 | 2 | 100.0% | 100.0% | 100% |
| 71 | Other | WNK |  | 14 | 14 | 93.6% | 93.6% | 93.6% | N/A | N/A | N/A | N/A | N/A |
| 72 | STE | MAP3K |  | 31 | 31 | 90.3% | 90.3% | 90.3% | 8 | 16 | 100.0% | 81.3% | 87.5% |
| 73 | STE | PAK |  | 116 | 116 | 89.7% | 93.9% | 91.8% | 46 | 92 | 89.1% | 87.0% | 87.7% |
| 74 | STE | PAKB |  | 14 | 14 | 92.1% | 90.0% | 91.1% | 9 | 18 | 100% | 88.9% | 92.6% |
| 75 | STE | PAKA |  | 90 | 90 | 91.1% | 92.9% | 92.0% | 37 | 74 | 94.6% | 93.2% | 93.7% |
| 76 | STE | YSK |  | 6 | 6 | 100.0% | 100.0% | 100.0% | 1 | 2 | 100.0% | 100.0% | 100% |
| 77 | STE | KHS |  | 7 | 7 | 74.3% | 84.3% | 79.3% | 6 | 12 | 66.7% | 66.7% | 66.7% |
| 78 | STE | MSN |  | 9 | 9 | 100.0% | 100.0% | 100.0% | 3 | 6 | 66.7% | 83.3% | 77.8% |
| 79 | STE | MST |  | 55 | 55 | 89.8% | 93.4% | 91.6% | 4 | 8 | 100.0% | 100.0% | 100% |
| 80 | STE | MAP2K |  | 101 | 101 | 78.9% | 82.5% | 80.7% | 19 | 38 | 63.2% | 73.7% | 70.2% |
| 81 | TK | Abl |  | 121 | 121 | 85.1% | 92.6% | 88.8% | 10 | 20 | 80.0% | 85.0% | 83.3% |
| 82 | TK | Ack |  | 10 | 10 | 91.0% | 91.0% | 91.0% | N/A | N/A | N/A | N/A | N/A |
| 83 | TK | Axl |  | 10 | 10 | 88.0% | 88.0% | 88.0% | N/A | N/A | N/A | N/A | N/A |
| 84 | TK | Csk |  | 29 | 29 | 89.6% | 93.1% | 91.4% | 7 | 14 | 71.4% | 100.0% | 90.5% |
| 85 | TK | DDR |  | 20 | 20 | 76.2% | 84.0% | 80.1% | N/A | N/A | N/A | N/A | N/A |
| 86 | TK | EGFR |  | 79 | 79 | 90.1% | 91.4% | 90.8% | 16 | 32 | 93.8% | 81.3% | 85.4% |
| 87 | TK | Eph |  | 32 | 32 | 96.6% | 94.5% | 95.6% | N/A | N/A | N/A | N/A | N/A |
| 88 | TK | FAK |  | 28 | 28 | 92.8% | 92.8% | 92.8% | 14 | 28 | 100.0% | 85.7% | 90.5% |
| 89 | TK | Fer |  | 14 | 14 | 92.8% | 92.8% | 92.8% | 5 | 10 | 80.0% | 100.0% | 93.3% |
| 90 | TK | Fes |  | 11 | 11 | 90.9% | 90.9% | 90.9% | N/A | N/A | N/A | N/A | N/A |
| 91 | TK | FGFR |  | 43 | 43 | 88.4% | 88.4% | 88.4% | 4 | 8 | 100.0% | 87.5% | 91.7% |
| 92 | TK | InsR |  | 51 | 51 | 91.6% | 88.6% | 90.1% | 15 | 30 | 93.3% | 83.3% | 86.7% |
| 93 | TK | IGF1R |  | 26 | 26 | 88.5% | 92.3% | 90.4% | 2 | 4 | 50.0% | 75.0% | 66.7% |
| 94 | TK | JakA |  | 59 | 59 | 88.3% | 90.1% | 89.2% | 30 | 60 | 76.7% | 85.0% | 82.2% |
| 95 | TK | TYK2 |  | 20 | 20 | 90.0% | 94.0% | 92.0% | N/A | N/A | N/A | N/A | N/A |
| 96 | TK | Met |  | 31 | 31 | 93.2% | 95.2% | 94.2% | 3 | 6 | 100.0% | 100.0% | 100% |
| 97 | TK | PDGFR |  | 43 | 43 | 92.1% | 90.2% | 91.1% | N/A | N/A | N/A | N/A | N/A |
| 98 | TK | Fms |  | 15 | 15 | 96.0% | 96.0% | 96.0% | N/A | N/A | N/A | N/A | N/A |
| 99 | TK | Kit |  | 8 | 8 | 100.0% | 100.0% | 100.0% | 1 | 2 | 0% | 100.0% | 66.7% |
| 100 | TK | Ret |  | 18 | 18 | 86.5% | 92.7% | 89.6% | 6 | 12 | 83.3% | 83.3% | 83.3% |
| 101 | TK | Brk |  | 22 | 22 | 94.9% | 95.0% | 95.0% | N/A | N/A | N/A | N/A | N/A |
| 102 | TK | Fgr |  | 9 | 9 | 100.0% | 94.4% | 97.2% | 2 | 4 | 50.0% | 75.0% | 66.7% |
| 103 | TK | Fyn |  | 66 | 66 | 85.9% | 90.2% | 88.0% | 25 | 50 | 84.0% | 88.0% | 86.7% |
| 104 | TK | HCK |  | 18 | 18 | 88.3% | 93.9% | 91.1% | 12 | 24 | 75.0% | 88.0% | 86.1% |
| 105 | TK | Lck |  | 64 | 64 | 92.0% | 93.2% | 92.6% | 24 | 48 | 87.5% | 87.5% | 87.5% |
| 106 | TK | LYN |  | 73 | 73 | 91.5% | 91.5% | 91.5% | 19 | 38 | 84.2% | 86.8% | 85.9% |
| 107 | TK | Src |  | 221 | 221 | 89.8% | 90.2% | 90.0% | 81 | 162 | 86.4% | 85.2% | 85.6% |
| 108 | TK | YES |  | 6 | 6 | 100.0% | 100.0% | 100.0% | 3 | 6 | 100.0% | 100.0% | 100% |
| 109 | TK | SYK |  | 61 | 61 | 94.5% | 93.3% | 93.9% | 8 | 16 | 100.0% | 87.5% | 91.7% |
| 110 | TK | ZAP70 |  | 21 | 21 | 91.4% | 93.3% | 92.4% | 8 | 16 | 87.5% | 87.5% | 87.5% |
| 111 | TK | Tec |  | 47 | 47 | 92.8% | 95.9% | 94.3% | 16 | 32 | 75.0% | 78.1% | 77.1% |
| 112 | TK | BTK |  | 19 | 19 | 89.5% | 89.5% | 89.5% | 4 | 8 | 100.0% | 100.0% | 100% |
| 113 | TK | ITK |  | 9 | 9 | 88.9% | 88.9% | 88.9% | 5 | 10 | 80.0% | 80.0% | 80.0% |
| 114 | TK | TXK |  | 8 | 8 | 100.0% | 100.0% | 100.0% | 2 | 4 | 0% | 100% | 66.7% |
| 115 | TK | Tie |  | 9 | 9 | 100.0% | 100.0% | 100.0% | 1 | 2 | 100.0% | 100.0% | 100% |
| 116 | TK | Trk |  | 17 | 17 | 89.4% | 96.4% | 92.9% | N/A | N/A | N/A | N/A | N/A |
| 117 | TK | VEGFR |  | 36 | 36 | 88.5% | 92.3% | 90.4% | N/A | N/A | N/A | N/A | N/A |
| 118 | TKL | IRAK |  | 9 | 9 | 100.0% | 92.9% | 96.5% | 2 | 4 | 50.0% | 100.0% | 83.3% |
| 119 | TKL | RAF |  | 9 | 9 | 66.7% | 77.8% | 72.3% | 13 | 26 | 53.8% | 65.4% | 61.5% |
| 120 | TKL | STKR Type1 |  | 17 | 17 | 91.2% | 91.2% | 91.2% | 14 | 28 | 71.4% | 78.6% | 76.2% |
| 121 | TKL | STKR Type2 |  | 11 | 11 | 98.9% | 99.6% | 99.3% | 3 | 6 | 66.7% | 66.7% | 66.7% |
| 122 | TKL | TGFbR |  | 20 | 20 | 94.0% | 95.0% | 94.5% | 9 | 18 | 77.8% | 66.7% | 70.4% |

Abbreviation: Sn, sensitivity; Sp, specificity; Acc, accuracy.

^a^The sizes of positive data and negative data are equal during the cross-validation processes. Ten sets of negative data are generated by randomly selecting from the non-phosphorylated residues of serine, threonine, and tyrosine in the phosphorylated proteins. Then, the cross-validation is performed for ten times to obtain an average accuracy for each kinase group.

^b^The independent testing data was mainly collected from PhosphoSitePlus, PHOSIDA, SysPTM, and HPRD. Several kinase-specific phosphorylation sites were manually curated from research articles. In order to make a feasible comparison with other published tools, the ratio of data size between positive set and negative set is 1:2 in independent testing data.
